# Low-Dose Alkylphenol Exposure Promotes Mammary Epithelium Alterations and Transgenerational Developmental Defects, But Does Not Enhance Tumorigenic Behavior of Breast Cancer Cells

**DOI:** 10.3389/fendo.2017.00272

**Published:** 2017-10-23

**Authors:** Clémence Chamard-Jovenin, Charlène Thiebaut, Amand Chesnel, Emmanuel Bresso, Chloé Morel, Malika Smail-Tabbone, Marie-Dominique Devignes, Taha Boukhobza, Hélène Dumond

**Affiliations:** ^1^CNRS-Université de Lorraine, UMR 7039, Centre de Recherche en Automatique de Nancy, BP70239, Vandoeuvre-lès-Nancy, France; ^2^Université de Lorraine, LORIA, UMR 7503, Vandoeuvre-lès-Nancy, France; ^3^Inria, Villers-lès-Nancy, France; ^4^CNRS, LORIA, UMR 7503, Vandoeuvre-lès-Nancy, France

**Keywords:** mammary gland, alkylphenol mix, cancer, development, estrogen receptor alpha 36

## Abstract

Fetal and neonatal exposure to long-chain alkylphenols has been suspected to promote breast developmental disorders and consequently to increase breast cancer risk. However, disease predisposition from developmental exposures remains unclear. In this work, human MCF-10A mammary epithelial cells were exposed *in vitro* to a low dose of a realistic (4-nonylphenol + 4-tert-octylphenol) mixture. Transcriptome and cell-phenotype analyses combined to functional and signaling network modeling indicated that long-chain alkylphenols triggered enhanced proliferation, migration ability, and apoptosis resistance and shed light on the underlying molecular mechanisms which involved the human estrogen receptor alpha 36 (ERα36) variant. A male mouse-inherited transgenerational model of exposure to three environmentally relevant doses of the alkylphenol mix was set up in order to determine whether and how it would impact on mammary gland architecture. Mammary glands from F3 progeny obtained after intrabuccal chronic exposure of C57BL/6J P0 pregnant mice followed by F1–F3 male inheritance displayed an altered histology which correlated with the phenotypes observed *in vitro* in human mammary epithelial cells. Since cellular phenotypes are similar *in vivo* and *in vitro* and involve the unique ERα36 human variant, such consequences of alkylphenol exposure could be extrapolated from mouse model to human. However, transient alkylphenol treatments combined to ERα36 overexpression in mammary epithelial cells were not sufficient to trigger tumorigenesis in xenografted Nude mice. Therefore, it remains to be determined if low-dose alkylphenol transgenerational exposure and subsequent abnormal mammary gland development could account for an increased breast cancer susceptibility.

## Introduction

Mammary duct network development initiates during fetal life and continues until first lactation in adulthood. Cellular and molecular data support the parallel between mammary gland morphogenesis processes and breast cancer initiation and progression (1, 2). Recent works indicate that fetal and/or neonatal exposure to xenoestrogens––such as bisphenol A (BPA)––alters the mammary gland development and subsequently drives an increased long-term risk of breast cancer (3, 4). Like BPA, long-chain alkylphenols are known to exert estrogen-like activities through binding to classical nuclear estrogen receptor alpha (ERα) and/or membrane-bound G protein-coupled estrogen receptor (GPER) (5). In 2004, the World Health Organization (WHO) published a nonylphenol NOAEL of 15 mg/kg/day for rat oral exposure and indicated that this value could be extrapolated to human (6). Indeed, previous studies by Chapin et al. (7) confirmed by Tyl et al. (8) demonstrated a lack of any transgenerational reproductive toxicity of dietary 4-nonylphenol (4NP) exposure during late gestation in Sprague–Dawley rats at doses ranging from 15 to 150 mg/kg/day. However, prenatal exposure to high doses of 4NP (25 mg/kg/day in MMTV-neu mice and 100 mg/kg/day in rats) causes altered development of the mammary gland, changes in steroid-receptor activation as well as increased synthesis of liver estriol (9, 10) in F1 pups. *In vitro*, 1 µM 4NP can also stimulate the proliferation of MCF-7 estrogen-sensitive breast cancer cells (11). More recently, Raecker et al. (12) indicated that 4NP and in a less-extent 4-tert-octyphenol (4tOP) are ubiquitously found in commercially available food intended for babies and toddlers. Based on consumption studies, the authors concluded that the infant intake of this mix ranges from 0.23 to 0.65 μg/kg/day, which is about 1,000 times less than the doses previously experimented before.

Recently, we have demonstrated that such a realistic dose of a 4NP:4tOP mixture can stimulate proliferation and modulate DNMT3L-dependent epigenetic status of testicular cancer germ cells (13). On the basis of this analysis, we assumed that a fetal exposure to low doses of alkylphenols could disturb male epigenetic imprinting establishment and drive male-inherited transgenerational alterations of reproductive organs. Indeed, such defects were described following fetal and/or neonatal exposure to environmental compounds, namely, BPA and vinclozolin (14). In the same study, we also demonstrated that the alkylphenol mix acts through a rapid, estrogen receptor alpha 36 (ERα36) dependent non-genomic pathway (13).

Estrogen receptor alpha 36 is a variant of the canonical human ERα lacking the helix 9–12 domain, likely to bind a great diversity of compounds (15). Recently, we showed that expression of human ERα36 transgene triggers neoplastic-like alteration of mice mammary gland (16). Since ERα36 can enhance expression of migration/invasion markers in normal epithelial cells, we wondered if its expression or activity could be stimulated under alkylphenol exposure, alter mammary epithelial cell differentiation, and consequently lead to developmental, structural, or functional disruption of the mammary gland.

First, we explored the molecular and cellular impact of a 4NP:4tOP mix exposure in the human MCF-10A mammary epithelial cell line by transcriptome and cell-phenotype analyses combined to functional and signaling network modeling. Our data point out ERα36 variant as a key node of the gene networks modulated by alkylphenols, involved in proliferation, migration, and apoptosis escape control. In order to investigate low-dose alkylphenol effect on the fetal and neonatal mammary gland morphogenesis, we set up a transgenerational assay of intrabuccal chronic exposure of wild-type or ERα36 transgenic (Tg) C57BL/6J P0 pregnant mice. Male-inherited exposure effects were assessed in F3 litters. The results strongly suggest that a low-dose alkylphenol exposure could promote a mammary gland abnormal development. Finally, we addressed if ERα36 overexpression in xenografted MCF-10A cells combined to systemic alkylphenol exposure would be sufficient to trigger mammary tumor formation in adult Nude mice.

## Materials and Methods

### Reagents

The test compounds, 4NP (CAS no. 84852-15-3) and 4tOP (CAS no. 140-66-9) were purchased from Sigma-Aldrich. 4tOP and 4NP were mixed based on their realistic concentration ratio (1:30) in infant food (12), thus forming the working mix called M4. In addition, 10-mM stock solutions (the concentration refers to 4NP in M4 mix) were prepared in either sesame oil (vehicle) for mice gavage or dimethylsulfoxide (DMSO) and further diluted in culture medium without phenol red for *in vitro* cell treatment. All working solutions were freshly made just before treatment and control cells were treated with DMSO, diluted with the same factor (1 × 10^−7^) as M4 and indicated as “vehicle” in the figures.

#### Animal Care and Use

##### Animals

Pathogen-free C57BL/6J mice (here considered as wt, Charles River Laboratories) or ERα36 Tg C57BL/6J mice (16) were maintained in temperature-controlled and light-controlled (10 h light, 14 h dark cycle) conditions in the Nancy Faculty of Pharmacy animal facility. All experimental procedures were approved by the French Minister of Research Committee for animal experiment (Protocol no. APAFIS#2168-2015110518268051 v5) in accordance with the Guide for Care and Use of Laboratory Animals. Cages, food, and bedding displayed negligible estrogenicity as tested by the supplier (Tecniplast); water was supplied from glass bottles only. Food (SAFE, France) and water were supplied *ad libitum*.

Transgenic C57BL/6J mice expressing a single copy of a human ERα36 transgene in the mammary gland under the control of the MMTV promoter were obtained as described in Thiebaut et al. (16). Tg males were mated with 7-week-old C57BL/6J wt females in order to obtain 50% hemizygotic Tg litters wherein the transgene is paternally inherited (Figure S1 in Supplementary Material). Parternal inheritance of the transgene was preferred to the maternal transmission in order to avoid a potential effect of the transgene expression during lactation. Before weaning, litters from wt females and Tg males were genotyped as previously described (16). In our study, wt litters issued from wt males and wt litters issued from hemizygote Tg males were compared.

##### Animal Treatment

Alkylphenol treatment composed of M4 dissolved in vehicle [0.05 μg/kg/day (D1), 0.5 μg/kg/day (D2), or 5 μg/kg/day (D3)] or either sesame oil (vehicle, D0) was administered to 7-week-old virgin C57BL/6J P0 females [5 P0 dams/dose 0 (D0) to dose 3 (D3)] from the first day of mating until delivery. Intrabuccal gavage was preferred to the stressful intragastric gavage which does not account for oral absorption (17), or to subcutaneously implanted minipumps which were reported to inappropriate for the delivery of estrogenic compounds to the mammary glands in rats (18, 19). These doses were thought to be environmentally relevant according to Raecker et al. (12). In order to avoid any hormonal interference with mammary gland development, we only used progeny from primipare C57BL/6J females, in spite of their tendency to cannibalism. In this work, the paternally inherited transgenerational effect of treatment can be measured in the F3 generation since F1 and F2 generations can be considered as directly exposed to the M4 mix in P0 mothers. At weaning (i.e., postnatal day 21, PND21), F1D0 to F1D3 wt or Tg litters as well as F2D0 to F2D3 or F3D0 to F3D3 paternally exposed wt or Tg litters were sacrificed by lethal intraperitoneal injection of 2 mg/kg pentobarbital (Sanofi/Aventis) and processed for further analyses.

### Cell Grafting in Nude Mice

Pathogen-free, 7-week-old athymic NMRI-nu (nu/nu) virgin female mice were purchased from Janvier Laboratories (Le-Genest-St-Isle, France). Animals were housed in the conditions described above, in the animal facility of the laboratory (CRAN, Nancy), with free access to water supplied from glass bottles and standard food *ad libitum*. Experimental procedures were approved by the French Minister of Research Committee for animal experiment (Protocol no. APAFIS#6158-2016072016446146 v5). As described above, sesame oil (vehicle) or 0.5 µg M4/kg/day treatment was administered by intrabuccal gavage, during 2 weeks before and 2 weeks after cell grafting. The dose of M4 was chosen based on significant results obtained in mammary ducts in C57BL/6J strain (see Results).

Orthotopic cell grafts were performed by a subcutaneous injection of a 3 × 10^6^ cells/50-μL phosphate-buffered saline (PBS) suspension, in the right mammary gland no. 4. The non-injected contralateral gland was used as a control. The following three mammary cell lines were used: ERα36 expressing MDA-MB-231 breast cancer line as a positive control for tumor formation, MCF-10A/Zeo as a non-cancerous negative control, or the previously described MCF-10A/ERα36 line overexpressing the human ERα36 receptor (16). Animals were sacrificed 4 weeks after grafting by lethal intraperitoneal injection of pentobarbital (see the above text).

### Mammary Gland Whole Mounts

The whole mounts were prepared by spreading the mammary glands onto glass slides and stained as described by Vandenberg et al. (20). A dedicated Matlab program for analysis of the mammary network, adapted from Tylcz et al. (21, 22), was previously described (16). The mammary network was quantified in terms of tree extension, branching, and amount of sprouts.

### Histology

To prepare paraffin sections, mammary glands were fixed with Davidson (23) for 24 h at room temperature, dehydrated, and embedded with paraffin (VWR). Moreover, 7-μm sections were cut on a Leitz rotary microtome (Leica) and mounted on Superfrost slides (Fisher Scientific); the entire mammary gland was sectioned at once. Sections were stained with hematoxylin/eosin/methyl green to determine the presence of epithelial cords. Sections with visible cords were used for further analysis.

#### Microarray Experiment

##### Sample Preparation and Data Normalization

Transcriptional profile analyses of MCF-10A cells exposed to 1 nM M4 for 1, 8, or 24 h were performed in triplicates on Affymetrix GeneChip U133 2.0 by the GenomEast Platform (IGBMC, Strasbourg, France). Biotinylated cRNA targets were prepared using the Ambion “MessageAmp™ Premier RNA Amplification Kit” according to the Instruction Manual P/N 4386269 Revision D (revision date: May 16, 2008), starting from 200 ng of total RNA extracted with RNeasy Mini Kit (Quiagen). Following fragmentation, 10 µg of cRNAs were hybridized for 16 h at 45°C, 60 rpm on Human GeneChip^®^ HG-U133 plus 2.0 arrays (Affymetrix). The chips were washed and stained in the GeneChip^®^ Fluidics Station 450 (Affymetrix) using the FS450_0004 script and scanned with the GeneChip^®^ Scanner 3000 7G (Affymetrix) at a resolution of 1.56 µm. Raw data (CEL Intensity files) were extracted from the scanned images using the Affymetrix GeneChip^®^ Command Console (AGCC) version 4.0. CEL files were further processed with Affymetrix Expression Console software version 1.3.1 to calculate probe set signal intensities, using Robust Multi-array Average (RMA) algorithms with default settings. Overall, 3,362 transcripts are differentially expressed compared to *t* = 0. Distinct kinetic variation pattern and transcript number in each pattern are described in Figure S2A in Supplementary Material. A total of 278 transcripts, 76 transcripts, and 2,743 transcripts were significantly up- or downregulated (absolute variation factor ≥2 in triplicate RNA samples, corrected *p*-value (*p* < 0.05) with Benjamini–Hochberg method) after 1-, 8-, or 24 h 1 nM M4 exposure, respectively. Differentially expressed genes (DEGs) were defined as genes whose expression is significantly affected by the M4 exposure, at any time of the kinetic (Figure S2B in Supplementary Material).

### Gene Ontology (GO) and Pathway Analyses

Gene ontology enrichment analyses were performed using the Panther online tool (Protein ANalysis THrough Evolutionary Relationships)^1^ (24). MSigDBv5.0 (Molecular Signature Database)^2^ (25, 26) was used to achieve the pathway enrichment analyses. The overlap between DEG list and (i) GO gene sets derived from the Biological Process part of GO (Table S1 in Supplementary Material) or (ii) Kyoto Encyclopedia Genes and Genomes (KEGG) pathways (Table S2 in Supplementary Material) were computed.

### Cell Culture

MCF-10A cells were purchased in 2015 from ATCC^®^ (CRL-10317) and maintained as described by Soule et al. (27). Briefly, cells were plated at a density of 2 × 10^4^ cells per well in 6-well plates in 5% HS-supplemented DMEM/F12 (GIBCO) medium for 24 h, then starved for 48 h in 2.5% charcoal-stripped HS-containing medium without phenol red. Treatments were performed on 24 h 0% HS-cultured cells.

### Transient Transfection and Stable Cell Lines Establishment

Stable MCF-10A cell lines transfected by pCDNA3.1-ERα36 or the empty expression vector were obtained as previously described (13) and named MCF-10A/ERα36 and MCF-10A/Zeo, respectively. The transfected cell lines were then subcloned and checked for ERα36 expression by western blot and reverse transcription polymerase chain reaction (RT-PCR) analyses before each experiment. MCF-10A/Zeo cells behaved similarly to the non-transfected MCF-10A cells in response to 1 nM M4 exposure whatever the assay performed (proliferation or migration or survival assay, data not shown).

### Real-time PCR Analysis

RT and real-time PCR analyses were performed as previously described (13). The following primers were used for qRT-PCR: *RPLPO* forward (Fw) 5′-GGCGACCGTGAAGTCCAACT-3′, *RPLPO* reverse (Rev) 5′-CCATCAGCACCACAGCCTTC-3′, *VIM* forward (Fw) 5′-GGGACGCAGACATCGTCATC-3′, *VIM* reverse (Rev) 5′-TCGTCATCGTCGAAATGGGC-3′, *CDH2* forward (Fw) 5′-ACAGTGGCCACCTACAAAGG-3′, *CDH2* reverse (Rev) 5′-CCGAGATGGGGTTGATAATG-3′. Assays were performed at least in triplicate, and the mean values were used to calculate expression levels, using the ΔΔC(*t*) method referring to *RPLPO* housekeeping gene expression.

### Crystal Violet Assay

The crystal violet assay was performed in 24-well plates. After each well was washed with PBS, the cells attached to the bottom of the plate were fixed and stained with 0.4% crystal violet solution in 2% ethanol for 30 min. After the plate was washed with water and dried, crystal violet was solubilized in 10% acetic acid and the absorbance at 570 nm was measured by a microplate reader (Victor x3, Perkin-Elmer).

### Flow Cytometry

After a 24 h 1 nM M4 or vehicle exposure, medium and adherent cells were harvested. Cells were washed twice with cold PBS, resuspended at 1.10^6^ cells/mL in PBS containing 4 µg/mL propidium iodide (PI) (P3566, Life Technologies), and 0.4 µg/µL RNAase A (Sigma-Aldrich) for 20 min at room temperature. PI fluorescence level was determined using the argon laser of a FACScalibur flow cytometer (Becton-Dickinson). For each sample, fluorescence intensity of 10,000 cells was analyzed using CellQuest Pro Software.

### Western Immunoblotting

Western blots were performed as described previously (13). The following primary antibodies were used: anti-P-Src Tyr416 (#6943P, Epitomics), anti-Cyclin B1 (#1495-1, Epitomics), anti-Cyclin D1 (#2922, Cell Signaling), anti-Cyclin E1 (CPA1171, Clinisciences), anti-Phospho-Rb ser 807/811 (#8516, Cell Signaling), anti-ERα36 (CY1109, Cell Applications), anti-P-Caspase 9 ser 196 (sc-11755, Santa Cruz Biotechnology), anti-P-Bad ser136 (sc-7999, Santa Cruz Biotechnology), anti-Bad (#9292, Cell Signaling), anti-PARP1 cleaved (552596, BD Pharmingen) and anti-Caspase 7 cleaved (#9494, Cell Signaling), and anti-Caspase 3 cleaved (#9664, Cell Signaling). The anti-β-Actin antibody (sc1615, Santa Cruz Biotechnology) or anti-glyceraldehyde-3-phosphate dehydrogenase (GAPDH, GTX100118, Genetex) were used as a loading controls. Protein expression profiles were revealed with Clarity Western ECL Substrate (Biorad) and banding quantification was performed using the Quantity One Chemidoc XRS software (Biorad).

### Immunofluorescence

Immunofluorescence was performed as described previously (13). The following primary antibodies were used: anti-NFκB (GTX102090, GeneTex), anti-β-catenin (E247, Epitomics #1247-s), anti-E-cadherin (GTX100443, GeneTex), and anti-N-cadherin (TA326835, OriGene). Goat anti-rabbit secondary antibody was coupled to AlexaFluor 555 (Invitrogen). Images were obtained with DS-Ri1 Nikon camera and Eclipse80i Nikon microscope and quantifications were performed using NIS-Elements BR 4.20.00 software (Nikon).

### TUNEL Assay

TUNEL assay was performed using the Apo-BrdU-IHC *in-situ* DNA fragmentation Assay kit (BioVision, USA) following the manufacturer instructions for the staining of cell preparations fixed on slides adapted for the use of AlexaFluor 555 (Invitrogen) goat anti-mouse secondary antibody. Nuclear DNA was stained with Hoechst (bisBenzimide H33342 Trihydrochloride, Sigma-Aldrich). Quantification of stained cell number was performed with NIS elements BR 4.20.00 software (Nikon).

### Scratch-Wound Assay

Scratch assays were performed using the Ibidi-culture inserts (Ibidi^®^/Biovalley) following the manufacturer instructions. Cultures were then washed to remove detached cells and debris and treated with vehicle or 1 nM M4. Quantification of wound mean diameter were performed at *t* = 0 and after a 6 h exposure by Phase-contrast image analysis with NIS-elements BR 4.20.00 software (Nikon).

### Statistical Analysis

Statistical analyses were performed with Matlab vR2014b software (MathWorks) by using Student’s *t*-test for unpaired samples and ANOVA followed by Bonferroni *post-hoc* test for multigroup comparison, with a significant *p*-value threshold below 5%. *p < 0.05; **p < 0.01; ***p < 0.001. SDs or SEs were indicated on figures as advocated by Altman and Bland (28).

## Results

### Microarray and Bioinformatic Analysis of MCF-10A DEGs after Kinetic Alkylphenol Exposure

In order to determine which cell function could be altered by alkylphenol exposure, we analyzed the transcriptional profile of MCF-10A cells exposed to M4. A kinetic microarray analysis of gene expression was performed after a 1-, 8-, or 24 h 1 nM M4 exposure. GO enrichment analysis of DEGs (Table S1 in Supplementary Material) indicated low levels of enrichment after 1 h M4 exposure, which underscored cell response to stress and metabolic processes. No GO biological process was found significantly enriched after an 8 h exposure, probably due to the limited number of DEGs. After a 24 h M4 exposure, the DEG list was enriched with genes mainly assigned to GO terms related to DNA structure and stability, cell cycle and proliferation, and splicing and cell death (Table S1 in Supplementary Material).

At each time point of the kinetic (1, 8, and 24 h), pathway enrichment analysis identified the maximal overlap between DEG lists and KEGG pathways for the “Pathways in Cancer” pathway. DEGs related to this pathway were mainly involved in the three cellular functions previously highlighted by the GO enrichment analysis: proliferation, evading apoptosis, and migration/invasion (Table S2 in Supplementary Material). Moreover, this analysis suggested that the main intracellular signaling pathways involved in M4 response were the MAPK, JAK-STAT, and P53-dependent ones (Table S2 in Supplementary Material). The accuracy of the *in silico* derived predictions was investigated by examining the MCF-10A cell phenotypes after M4 exposure.

### Focus on Cell Cycle

**Figure 1:**
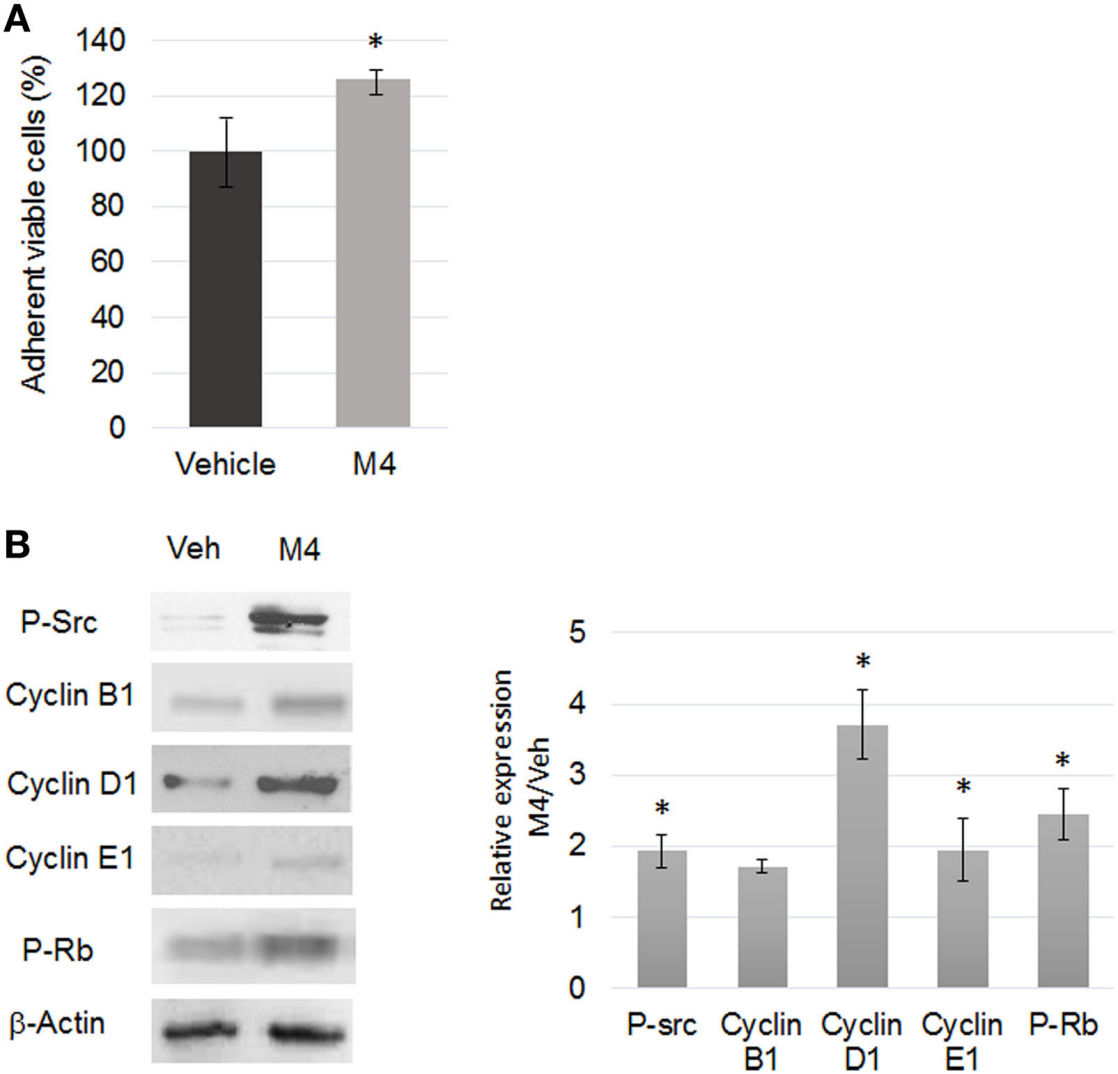
M4 stimulating cell proliferation. **(A)** Quantification of MCF-10A cell viability by crystal violet assay after 24 h vehicle or 1 nM M4 exposure. M4 treatment triggered a 26% increase of cell proliferation. Each bar represents mean ± SD. *N* = 3. *p < 0.05. **(B)** Western blot analyses of protein expression level (left) and corresponding quantifications (right) of phospho-Src (tyr416), Cyclin B1, D1, E1 and phospho-Rb (retinoblastoma protein) in 24 h vehicle or 1 nM M4-treated cells. β-Actin was used as a loading control. Enhanced Src and Rb phosphorylation as well as Cyclin D1 and Cyclin E1 expression was observed under M4 treatment. Each bar represents mean ± SD. *N* = 4. *p < 0.05.

Expression of key genes and corresponding proteins involved in cell cycle control and regulation was determined by western blot analysis after a 24 h M4 exposure. Quantification of cell viability by crystal violet staining indicated a 23 ± 0.5% augmentation after M4 versus vehicle 24 h exposure (*p* < 0.05, Figure 1A). Flow cytometry analyses confirmed that a 24 h M4 exposure triggered cell cycle entry even if the data were not statistically significant (66 ± 1.3% G0/G1 phase in vehicle exposed cells versus 60 ± 0.8% G0/G1 phase in M4 exposed cells; *N* = 3). Moreover, the expression of more than 25 genes involved in all cell cycle steps was modified by alkylphenol exposure (Table 1), all of them sustaining enhanced cell cycle entry or progression.

**Table 1:** List of genes associated with the KEGG pathway “Cell cycle” and corresponding fold expression variation after 24 h M4 exposure as measured in transcriptomic analyses.

Gene symbol	Fold expression variation 24 h–M4/Veh
BUB1	10.85
BUB1B	16.43
CCNB1	11.11^a^
CCNB2	2.33
CCND1	4.61^a^
CDC20	5.44
CDC25A	6.85
CDC25C	5.47
CDC6	24.60
CDC7	5.20
CDK2	2.33
CDK4	2.05
CDKN2C	4.85
E2F2	2.98
MCM2	6.85^a^
MCM3	8.77
MCM4	5.91
MCM5	5.19^a^
MCM6	2.11
MCM8	2.51
MCM10	9.09^a^
PCNA	3.58
PRKDC	2.12
RBL1	2.96
SKP1	2.18
TGFB2	2.18
TTK	17.24

*^a^Fold expression variation that was validated by real-time PCR analyses (not shown)*.

*KEGG, Kyoto Encyclopedia Genes and Genomes*.

Figure 1B shows that Src phosphorylation, Cyclin D1 and Cyclin E1 expression as well as Retinoblastoma (Rb) protein phosphorylation were significantly induced (*p* < 0.05). According to the microarray analysis, almost all genes encoding the mini chromosome maintenance (MCM) protein family involved in proper positioning of DNA replication forks as well as DNA mismatch repair protein encoding genes, MSH2 and MSH6, were upregulated after M4 exposure (Figure S3A in Supplementary Material). Therefore, MCF-10A cell line exposed to M4 or not was also tested for random amplification of polymorphic DNA (RAPD): among the 10 GC-rich decamer primers randomly designed to fingerprint the genomic DNA [Figure S3B in Supplementary Material (29, 30)], 50% displayed an altered profile after M4 exposure, suggesting the outbreak of genomic instability (Figure S3C in Supplementary Material). Taken together, these results supported the GO enrichment prediction that M4 may stimulate cell cycle despite non-reliable DNA replication initiation, thus altering genomic stability.

### Focus on Apoptosis Escape

**Figure 2:**
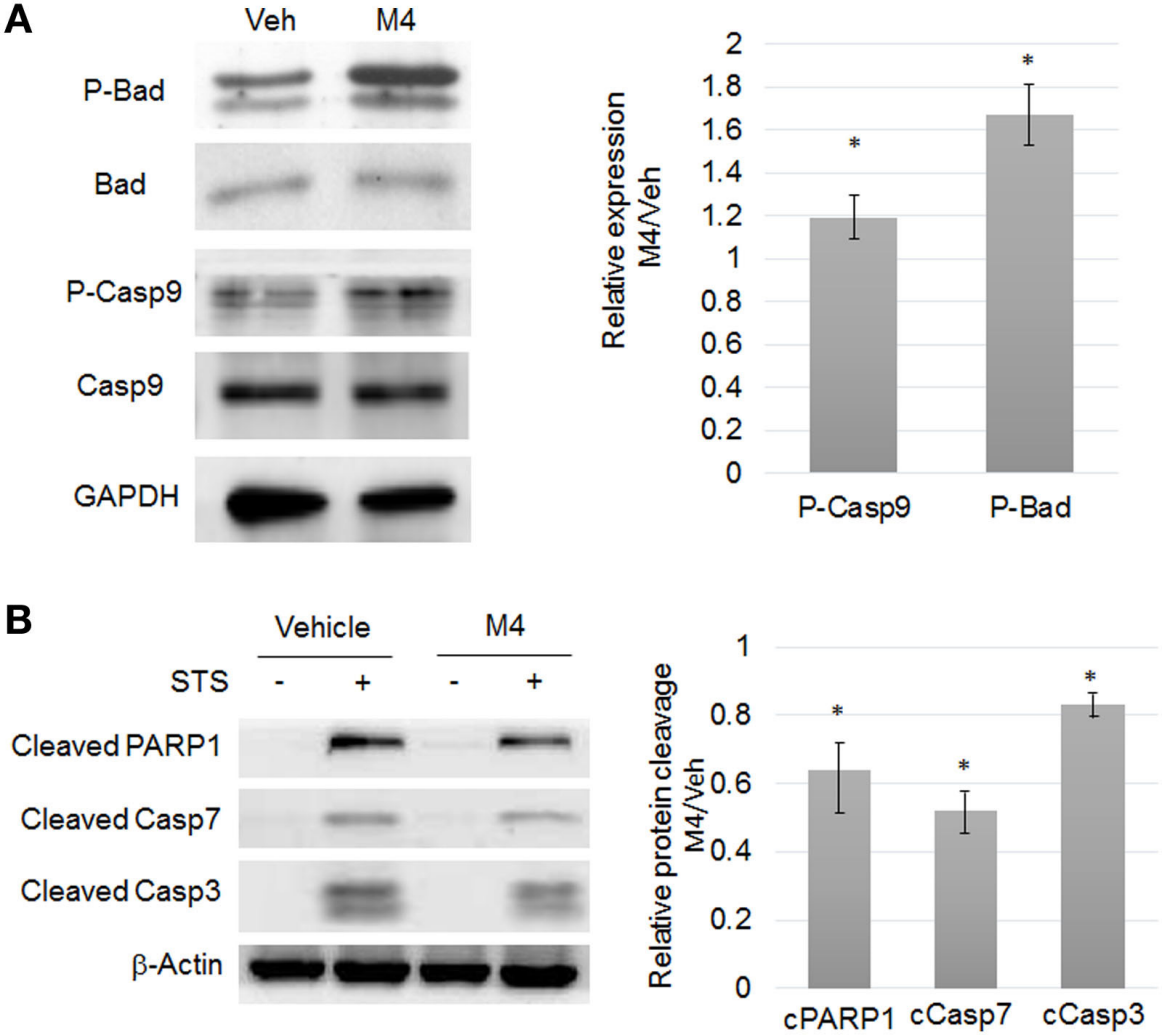
M4 stimulating resistance to apoptosis. **(A)** Western blot analyses of protein expression level (left) and corresponding quantifications (right) of phospho-Bad (ser136), phospho-Caspase 9 (ser196) in 24 h vehicle or 1 nM M4-treated cells. M4 treatment triggered a significant 19% and 67% increase of Bad (ser136) and Caspase 9 (ser196) phosphorylation, respectively. Each bar represents mean ± SD. *N* = 3. *p < 0.05. **(B)** MCF-10A cells were pretreated for 24 h with vehicle or 1 nM M4, and then exposed to 0.25 µM staurosporine (STS) for 6 h or not (Veh). Cleavage of PARP1, Caspase 7, and Caspase 3 were evaluated with specific antibodies. β-Actin was used as a loading control. Results are represented as M4 + STS/STS ratio. M4 treatment triggered a significant 36, 48, and 17% decrease of PARP1, Caspase 7, and Caspase 3 cleavage, respectively. Each bar represents mean ± SD. *N* = 4. *p < 0.05.

MCF-10A cells were exposed to DMSO or M4 for 24 h and then treated for 6 h by either vehicle or staurosporine (STS). M4 alone triggered Bad (ser136) and Caspase 9 (ser196) enhanced phosphorylation (*p* < 0.05; Figure 2A), suggesting that those proteins became protected against pro-apoptotic intracellular signals and NF-κB nuclear translocation (Figure S5A in Supplementary Material). Co-treatment with M4 and STS led to 17 ± 2% reduction Caspase 3, 48 ± 6% reduction of Caspase 7, and 36 ± 5% of PARP1 cleavage compared to STS alone (*p* < 0.05; Figure 2B). This apoptosis resistance triggered by M4 was confirmed by the observation of cytochrome c release and nuclear fragmentation measurement which was reduced by 55 ± 3% (*p* < 0.05; data not shown).

### Focus on EMT

**Figure 3:**
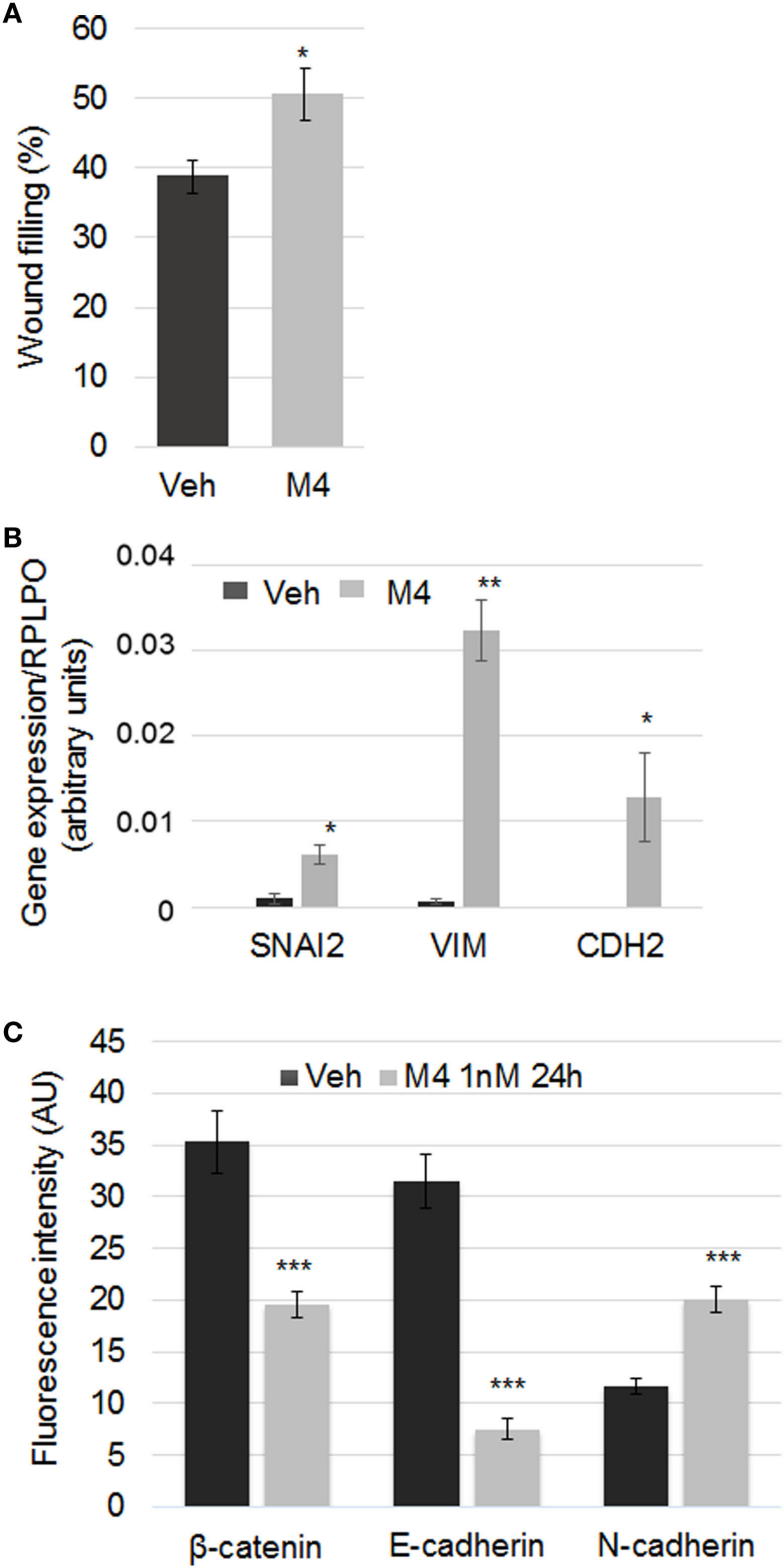
M4 promoting epithelial-mesenchymal transition (EMT). **(A)** A wound was performed on a confluent monolayer culture of MCF-10A cells. The histogram depicts the wound width when measured after a 6 h treatment. Each bar represents mean ± SD. *N* = 5. *p < 0.05. **(B)** MCF-10A cells were treated for 24 h with vehicle or 1 nM M4. Gene expression of EMT markers was measured by RT-PCR analysis. SNAI2, VIM, and CDH2 expression was induced after M4 exposure. The housekeeping gene *RPLPO* was used as the reference gene. Each bar represents mean ± SD. *N* = 3. *p < 0.05; **p < 0.01. **(C)** MCF-10A cells were treated for 24 h with vehicle or 1 nM M4. Quantification of cell–cell adhesion protein expression was performed by immunofluorescence with specific antibody (red, AlexaFluor 555). An “E- to N-cadherin switch” as well as a significant decrease of β-catenin expression was observed in M4-treated MCF-10A cells as compared to control. Average fluorescent signal intensities were quantified from at least five cells per condition in each experiment (see Materials and Methods). Each bar represents mean ± SD. *N* = 4. ***p < 0.001. RT-PCR, reverse transcription polymerase chain reaction.

Using the scratch-wound assay, we observed that M4 exposure of MCF-10A cells triggered an accelerated closure of the wound created in a confluent monolayer culture (*p* < 0.05; Figure 3A; Figure S4 in Supplementary Material). Therefore, expression of epithelial-mesenchymal transition (EMT) markers was analyzed by real-time PCR. SNAI2 (*p* < 0.05), VIM (*p* < 0.01), and CDH2 (*p* < 0.05) expression were induced after M4 exposure (Figure 3B). Expression and localization of proteins known to be involved in cell–cell junctions were also examined: a list of genes associated with the KEGG pathway “Biological adhesion” and corresponding fold expression variation after 24 h M4 exposure was extracted from the transcriptomic data (Table 2). The expression of those 65 genes was repressed after M4 treatment. M4 treatment also triggered the decrease of beta-catenin (43%; *p* < 0.001) and E-cadherin (78%; *p* < 0.001) membrane immunofluorescence staining, whereas N-cadherin staining was augmented by 66% (Figure 3C; Figure S5B in Supplementary Material).

**Table 2:** List of genes associated with the GO term “Biological adhesion” and corresponding fold expression variation after 24 h M4 exposure as measured in transcriptomic analyses.

Gene symbol	Fold expression variation 24 h–M4/Veh	Gene symbol	Fold expression variation 24 h–M4/Veh
ADAM17	0.44	IL7R	0.17
ANTXR1	0.36	ITGA10^a,b,c^	0.45
ANXA1	0.45	ITGA5^a,b,c^	0.49
ARHGEF7^b^	0.45	ITGAM^b^	0.28
ATP2A2	0.39	ITGB4^a,b,c^	0.36
AZI2	0.41	ITGB8^a,b,c^	0.46
B4GALT1	0.41	KIRREL	0.41
BCL2L11	0.31	LAMA3	0.38
CD164	0.50	LAMA5	0.41
CD36^a^	0.09	LAMC1^a,c^	0.37
CD44^a^	0.41	LEPR	0.23
CD47^a^	0.39	LMLN	0.40
CD99	0.42	LOXL2	0.16
CFDP1	0.46	LY6D	0.32
CLCA2	0.41	MICALL2	0.32
CLEC7A	0.17	MPZL2	0.32
COL12A1	0.48	MPZL3	0.33
COL7A1	0.29	NT5E	0.38
CTNNA1	0.47	OLR1	0.47
CTTN	0.31	PCDH7	0.48
CYR61	0.42	PCDH9	0.33
DLG1	0.47	PCDHGA4	0.31
DST	0.21	PDZD2	0.28
EGFR^b,c^	0.25	PKN2	0.40
ENG	0.48	PRKCE	0.40
FAT2	0.43	PTPRK	0.37
FN1^a,b,c^	0.26	SCARB1	0.35
GNAS	0.37	SLC7A11	0.19
GPNMB	0.14	SRGAP2	0.30
HAS3	0.36	SRPX2	0.31
HPSE	0.29	WISP2	0.25
IGFBP7	0.40	WNT4	0.32

*Genes that were also associated with KEGG pathways “ECM-receptor interaction” ^a^ and/or “Regulation of actin cytoskeleton” ^b^ and/or “Focal adhesion” ^c^ are indicated*.

*GO, gene ontology; KEGG, Kyoto Encyclopedia Genes and Genomes*.

### Involvement of ERα36 in M4-Dependent Pathways in MCF-10A Cells

**Figure 4:**
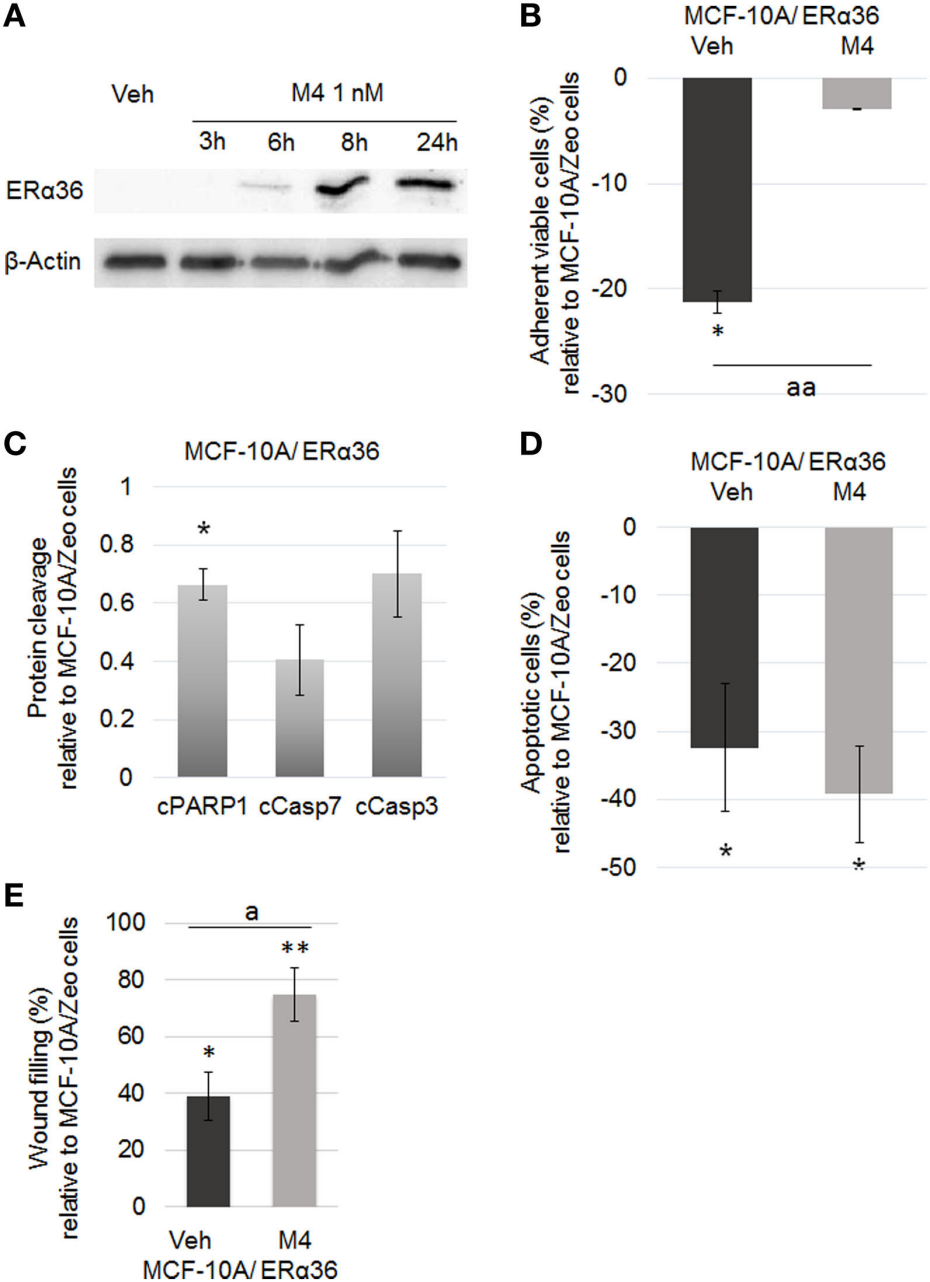
ERα36 overexpression enhancing M4-dependent migration but not proliferation or apoptotic escape. **(A)** Representative western blot of a M4 kinetic treatment of the MCF-10A cell line. M4 (1 nM) stimulated ERα36 protein expression. β-Actin was used as a loading control. *N* = 3. **(B)** Quantification of MCF-10A/ERα36 cell viability by crystal violet assay after 48 h vehicle or 1 nM M4 exposure. Histogram depicts the percentage of MCF-10A/ERα36 viable cells compared to MCF-10A/Zeo cells after treatment. M4 triggered a significant 18. 4% increase of MCF-10A/ERα36 cell proliferation rate. Each bar represents mean ± SD. *N* = 5. * = significantly different from MCF-10A cells. aa = significantly different from MCF-10A/ERα36 vehicle treated cells (*p < 0.05; aa *p* < 0.01). **(C)** MCF-10A/Zeo or MCF-10A/ERα36 cells were exposed to 0.25 µM staurosporine (STS) for 6 h. Quantification of cleaved Caspase 3 (cCasp3), Caspase 7 (cCasp7) or PARP1(cPARP1) levels showed that ERα36 overexpression triggered a reduction of apoptotic markers. Each bar represents mean ± SD. *N* = 3. *p < 0.05. **(D)** MCF-10A cells were pretreated for 24 h with vehicle or 1 nM M4, and then exposed to 0.25 µM STS for 24 h. The percentage of apoptotic cells was assessed by TUNEL assay. Results were represented as MCF-10A/ERα36 versus MCF-10A/Zeo cells ratio. No significant additive effects of ERα36 overexpression and M4 treatment were observed concerning apoptosis escape. Each bar represents mean ± SD. *N* = 5. *p < 0.05. **(E)** A wound was performed on a confluent monolayer culture of either MCF-10A/Zeo or MCF-10A/ERα36 cells pretreated for 48 h with vehicle or 1 nM M4. Histogram depicts the percentage of wound healing for MCF-10A/ERα36 versus MCF-10A/Zeo cells after 6 h. Results indicated that M4 treatment enhanced the migratory potential of MCF-10A/ERα36 cells. Each bar represents mean ± SD. *N* = 3. * or ** = significantly different from MCF-10A cells. a = significantly different from MCF-10A/ERα36 vehicle treated cells (* or a: *p* < 0.05; **p < 0.01). ERα36, estrogen receptor alpha 36.

Microarray analysis indicated that none of the potential estrogen receptors (ERα, ERβ, GPER) expression was affected by M4 treatment. Since alkylphenol are described as estrogen mimicking compounds, we focused on the variant ERα36 whose expression was stimulated 8-fold by 24 h M4 exposure (Figure 4A). The potential additive effects of ERα36 overexpression and M4 treatment were assessed in the MCF-10A/ERα36 cell line which stably overexpressed ERα36 compared to MCF-10A/Zeo, both exposed to vehicle or M4. Quantification of cell viability by crystal violet staining indicated a marked reduction in MCF-10A/ERα36 cell division rate compared to MCF-10A/Zeo (16). Nevertheless, MCF-10A/ERα36 proliferation remained inducible by M4 exposure in the same range (18% versus 23%) as MCF-10A/Zeo ones (*p* < 0.01; Figure 4B). MCF-10A/ERα36 and MCF-10A/Zeo were exposed for 24 h to vehicle or M4 and then treated with STS as described above. The quantifications of Caspase 3, Caspase 7, and PARP1 cleavage in both cell lines indicated that ERα36 overexpression led to a significant 34% reduction of PARP1 cleavage (*p* < 0.05), whereas a non-significant trend toward cleavage moderation was also observed for Caspase 7 and Caspase 3 (Figure 4C). A TUNEL assay also indicated that ERα36 overexpression was sufficient to reduce the number of MCF-10A/ERα36 apoptotic cells compared to MCF-10A/Zeo ones after STS exposure (*p* < 0.05; Figure 4D). Nevertheless, M4 pre-treatment of STS exposed MCF-10A/ERα36 cells did not significantly potentialize resistance to apoptosis. Finally, scratch-wound assays performed on MCF-10A/Zeo or MCF-10A/ERα36 indicated that ERα36 overexpression enhanced M4-stimulated migration potential of mammary epithelial cells (*p* < 0.01; Figure 4E; Figure S4 in Supplementary Material).

### *In Vivo* Assessment of Developmental and Transgenerational Impact of Alkylphenol Exposure

We first addressed the effects of maternal exposure to dietary relevant levels of long-chain alkylphenol M4 mix on fetal mammary gland development (see Materials and Methods). Whole mount analyses of mammary gland from F1 litters sacrificed at weaning (PND21) showed no significant mammary tree total extension or duct branching number in M4 maternally exposed F1 pups when compared to vehicle exposed ones. However, premature lumen opening (*p* < 0.01) and epithelium (*p* < 0.05) thickening (*p* < 0.05) were observed (Figure S6 in Supplementary Material).

**Figure 5:**
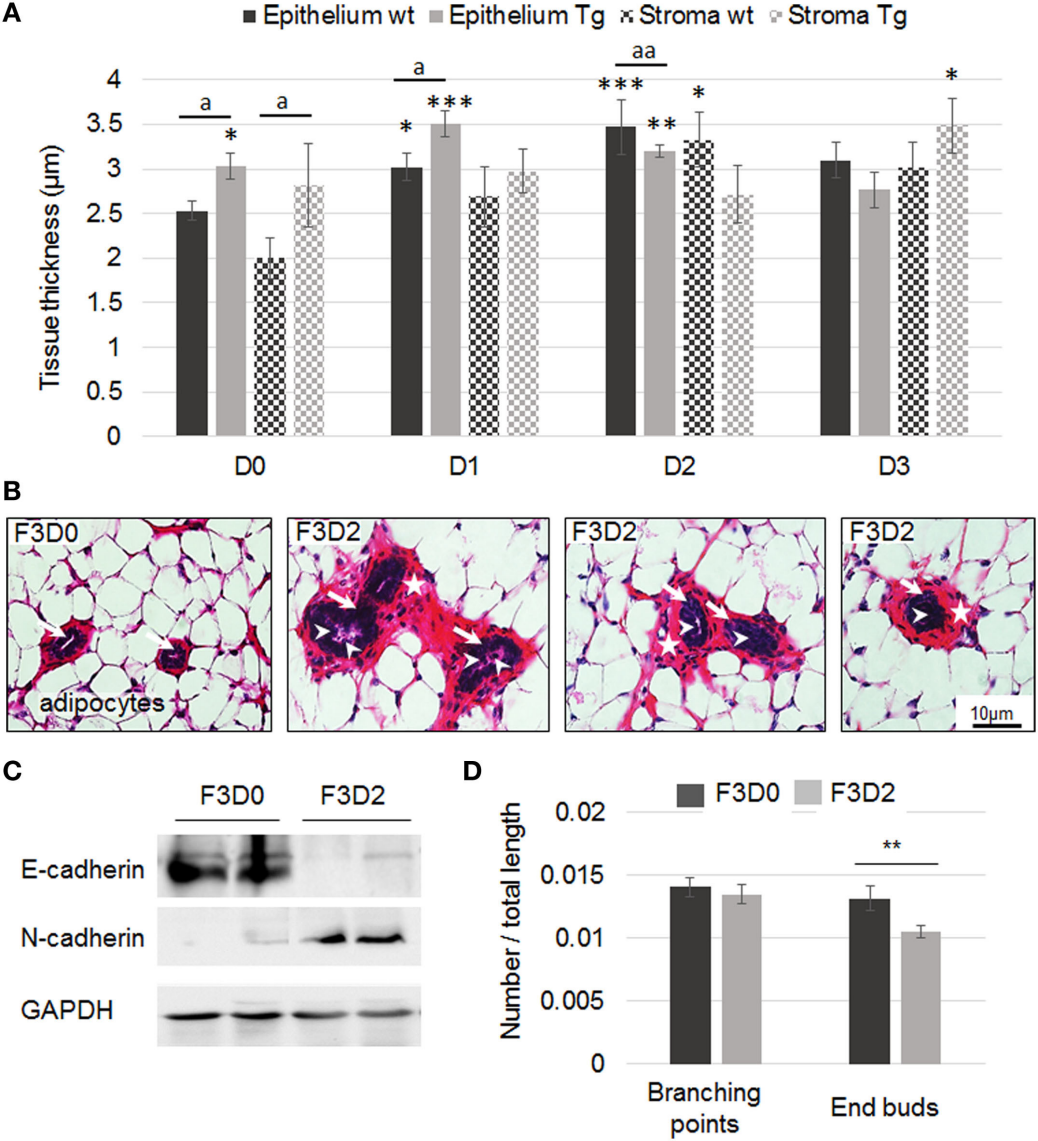
Mammary gland histology of F3 wild-type or transgenic (Tg) mice exposed to M4. **(A)** Quantification of epithelium and stroma thickness in F3 mammary gland following exposure to sesame oil (D0) or M4 [0.05 µg (D1), 0.5 µg (D2), or 5 µg M4/kg/day (D3)]. Measurements were performed on PND21. Each bar represents mean ± SEM. Epithelium or stroma thickness was measured on at least five independent slices from each animal included in the experiment. N: number of animals/dose/genotype. D0wt: *N* = 7; D0Tg: *N* = 5; D1wt: *N* = 6; D1Tg: *N* = 4; D2wt: *N* = 4; D2Tg: *N* = 8; D3wt: *N* = 3; D3Tg: *N* = 4. *, **, or *** = significantly different from D0 wild-type (wt) mice. a or aa = Tg mice significantly different from wild-type littermates exposed to the same dose (* or a *p* < 0.05; **; aa *p* < 0.01; ***p < 0.001). **(B)** F3 mammary gland exposed to M4 (D2 = 0.5 µg/kg/day). The slices show mammary ducts surrounded by stroma (stars) and adipocytes. Disorganization of the epithelium (arrows) was observed in D2 exposed F3 mammary glands and cellular material (arrowheads) was present into the lumen of some F3D2 female ducts. Scale bar, 10 µm. **(C)** Representative western blot analyses of E-cadherin, N-cadherin, and GAPDH (loading control) protein expression levels in total mammary gland harvested from F3D0 or F3D2 pups. **(D)** Mammary trees from F3D0 (*N* = 4) or F3D2 (*N* = 9) exposed mice were whole mounted. Computational analysis of the mammary tree total extension, number of branching or end buds was performed using dedicated software. Results are represented as a ratio of number of branching or end buds/mammary tree total length. The number of end buds was significantly reduced in F3D2 pups. Each bar represents mean ± SEM. **p < 0.01. GAPDH, glyceraldehyde-3-phosphate dehydrogenase.

The second part of the *in vivo* study used a transgenerational model of M4 paternally inherited exposure (Figure S1 in Supplementary Material). F3 mammary gland were harvested at weaning and submitted to histological analysis. A non-monotonic dose response to M4 exposure was observed in terms of epithelium and stroma thickening. The maximal impact was measured for the dose 2 (0.5 µg/kg bw/day) which augmented epithelium and stroma thickness by 38% (*p* < 0.01) and 60% (*p* < 0.05), respectively (Figure 5A). Moreover, more than 80% of F3D2 mammary ducts displayed non-cohesive epithelium, cellular material into the lumen or cribriform patterns (Figure 5B). At the molecular level, we observed a loss of E-cadherin along with the induction of N-cadherin expression (Figure 5C). Finally, mammary tree whole mount computational analyses indicated a trend toward an increase of mammary tree total extension (F3D0: 4,515 ± 108 μm, F3D2: 5,272 ± 83 μm; *p* = 0.073; *N* = 9; Amand Chesnel, unpublished data). A significant 21% reduced number of end buds (*p* < 0.01), whereas no branching point number variation in F3D2 pups were observed (Figure 5D).

### M4 Exposure of Transgenic Mice Expressing a Human ERα36 Transgene in the Mammary Gland

Then, we addressed the combined effects of M4 exposure and human ERα36 expression in the mammary gland of mice transgenerationally exposed to M4, as described above for the wild-type mice. Epithelium thickness was affected by both the transgene expression and the treatment as a non-monotonic dose–response relationship: compared to wt animals, the transgene expression seemed to enhance the treatment consequences for D1 (*p* < 0.05) but conversely to lower this effect for D2 (*p* < 0.01) and D3 (Figure 5A). The stroma displayed a similar but statistically non-significant profile (Figure 5A).

At adulthood, we observed a non-significant trend to epithelium thickening in F3 control, D1- or D2-treated Tg mice but a particular phenotype resembling the one depicted at weaning for D3-treated animals (Figure S7 in Supplementary Material). No combined effect of human ERα36 expression and M4 exposure was detected for either the epithelium or the stroma.

### ERα36 Overexpression in M4 Exposed Xenografted Nude Mice

In order to address the ability of M4 treatment combined to ERα36 overexpression to trigger carcinogenesis of the adult mammary gland, we orthotopically grafted the MCF-10A cell line, either expressing or not the ERα36 receptor. No tumor was observed in MCF-10A/Zeo or MCF-10A/ERα36 grafted mice whatever the treatment. Conversely, 80% of MDA-MB-231 grafted mice developed a tumor 5 days after cell injection. Once gavage was stopped, tumor volume was slightly increased in MDA-MB-231 grafted mice previously exposed to M4 compared to vehicle treated ones. At end point, tumor volume and histology were similar in M4 or vehicle-exposed animals (Figure S8 in Supplementary Material).

## Discussion

The data presented herein support for the first time a deleterious impact of exposure to low doses of the M4 alkylphenol realistic mixture through P0 maternal exposure followed by a second-generation male transmission. Instead, most studies available depict the direct consequences of several milligrams of either 4NP or 4tOP, separately. The study by Moon et al. (10) indicated that Long Evans rats exposed to 100 mg/kg during gestation days 15–19 displayed advanced lobular development of their mammary gland on PND21, while the glands from the “low” dose (10 mg/kg) were unaffected. The conversion of immature to mature structures was also reported in Noble rats in response to 7.1 mg/24 h or 0.01 mg/24 h nonylphenol exposure (31). In our study, PND21 F1 pups, fully exposed to a maximal dose of 5 µg/kg M4 through gestation exhibited mammary gland architecture alterations but no precocious differentiation or extension of the mammary tree. Nevertheless, Colerangle and Roy (31) showed a significant stimulation of cell cycle and proliferation of mammary epithelial cells (24 and 38% for the low and high exposure, respectively) which correlates to epithelium thickening observed in our F1 mammary gland samples and the 23% accelerated proliferation combined to cyclin up-regulation observed in MCF-10A cells.

While testing the combined effects of M4 exposure and ERα36 expression on tumor initiation, we performed a positive control group in which the Nude mice, xenografted with ERα36 expressing MDA-MB-231 breast tumor cells, were gavaged with sesame oil or M4 (dose 2). Mice developed a tumor without any difference between vehicle and M4 treated animals in terms of tumor latency, multiplicity of histology. Nevertheless, we observed a trend to tumor growth rate enhancement in mice exposed to alkylphenols, which suggest that such an exposure could be considered as a tumor promoting environment (= boost tumor growth). This model is quite different from MMTV-erbB2 Tg mice used by Jenkins et al (32) or the carcinogen DMBA (dimethylbenz[a]anthracene) induced tumorigenesis described by Betancourt et al. (33) in which both teams demonstrated a pro-tumoral effect of BPA exposure. Opposite results were obtained by Peng et al. (34), who reported a protective effect of a 40 mg/kg octylphenol exposure in DMBA-treated Sprague–Dawley rats. Nevertheless, these data suggest that a transient direct exposure to alkyphenols at adulthood is not sufficient by itself to trigger mammary carcinogenesis. Indeed, Sprague et al. (35) found no relationship between breast density of postmenopausal women and blood concentration of either nonyl- or octylphenol.

Since lifetime exposure to alkylphenols is the most common feature for human populations in western countries, we addressed the transgenerational male transmission of mammary gland abnormalities, especially for a dose of 0.5 µg/kg/day, representative of human exposure (12). F3 pups from P0-treated dams, which were not directly exposed to the M4 mixture, displayed paternally inherited elevated lumen diameter, epithelium, and stroma thickness as well as end bud number when compared to F3 originating from untreated dams. The main phenotype was the presence of numerous stoppered mammary ducts which display either cellular material scattered into the lumen or several septa and epithelium misorganization. These phenotypes could be interpreted as a partial disruption of stroma-epithelium interactions (36) as confirmed by severe reduction of E-cadherin and stimulation of N-cadherin expression in F3 pups originating from animals exposed to M4 *in utero*. Such nonylphenol-dependent disruption of cell–cell adherence and epithelial structure was also observed in rat seminiferous tubules after high-dose exposures (37). The mixed phenotypes observed in mammary glands from this study resemble ductal atypia rather than intraductal hyperplasia described by Vandenberg et al. (38) in CD-1 mice exposed to BPA. In a review, Paulose et al. (36) also listed systemic, tissular, cellular, and molecular alterations generated in adult mice by BPA perinatal exposure. Namely, the authors describe a partial and genome-wide modification of DNA methylation (36). Even if we did not observe any histological alteration in adult mice, we detected *in vitro* significant M4-dependent changes in ERα36 promoter methylation status (data not shown), suspected to be linked to the protein expression level. Taken together, our data suggest that, like BPA, M4 exposure could have multiscale consequences on mammary gland development. Therefore, it would be of great interest to follow aging exposed mice compared to control ones to address the hypothesis of any alkylphenol-dependent fetal programming of adult disease.

To address the molecular pathways that could be involved in alkylphenol-dependent mammary epithelial cell alteration in human, we performed a comprehensive analysis of gene expression under alkylphenol *in vitro* exposure of the human MCF-10A cell line. GO and KEGG pathway enrichment analysis confirmed by *in vitro* experiments indicated that the main functions affected by the treatment––i.e., proliferation, survival, and migration- could ultimately result in cell transformation. Indeed, perturbation of cell cycle and enhanced proliferation concomitant with genetic instability are considered as a risk factor for the development of cancer. Nonylphenol-dependent stimulation of clastogenic and mutagenic mechanisms as well as enhanced cell cycle and proliferation has been reported many times in different *in vitro* and *in vivo* models (31, 39–41).

Our data reporting an enhanced survival to STS-induced apoptosis in MCF-10A are in line with inhibition of induced cell death by BPA or nonylphenol reported in rat PC12, hippocampal, and neuronal cells (42, 43). Another consequence of alkylphenol exposure was the stimulation of migratory potential as supported by enhanced wound filling and EMT marker expression. These *in vitro* data point in the same direction as mammary epithelium morphological changes observed *in vivo*. Similar morphological and transcriptomic results were obtained in low-dose BPA-exposed mice which show ductal alterations correlated to induction of anti-apoptotic and reduction of focal adhesion-related genes (44).

Alkylphenols are known to bind estrogen receptors with a relative binding affinity to 17β-estradiol in the range of 0.1% for ERα and 50% for GPER (5, 45). In order to understand whether the molecular and global phenotypes observed after alkylphenol exposure were due to their estrogenicity and could be extrapolated to human, we turned back to the microarray analysis of human normal epithelial MCF-10A cells but observed that none of the probe covering known nuclear or membrane estrogen receptor genes displayed differential expression at any time. Since KEGG pathway enrichment pointed to JAK-STAT and MAPK signaling pathways, we assumed that the ERα36 variant could be involved in M4 response. Indeed, ERα36 was previously demonstrated to trigger (i) STAT3 signaling in mammary epithelial cells, (ii) MAPK signaling in breast cancer cells, and (iii) alkylphenol-dependent response in seminoma cells (13, 16, 46, 47). Recently, we also demonstrated that ERα36 overexpression in MCF-10A cells or mammary gland is sufficient, by itself, to alter normal breast epithelial phenotype *in vitro* and *in vivo* (16). Therefore, we showed that ERα36 expression was induced by M4 exposure in mammary epithelial cells and addressed the consequences of an alkylphenol treatment in epithelial cells displaying a forced ERα36 expression. An additive effect of both conditions was only observed for the enhanced migration phenotype of MCF-10A cells. This suggests that alkylphenols modulate (i) proliferation and survival in an ERα36 dose-independent manner and (ii) migration in an ERα36 dose-dependent way, probably through a combination of cofactor association and transduction pathway activation. Therefore, ERα36 expression level could be a promising marker of breast epithelium leakage in the context of endocrine disruptor exposure. Similar results were obtained from breast cancer patient samples, in which we and others demonstrated that ERα36 expression level is a relevant predictor of metastatic progression (17, 46, 48) but to our knowledge, this is the first description of a functional role of ERα36 in normal epithelial mammary cells exposed to xenoestrogens. Therefore, ERα36-dependent modifications of cell phenotype that could be related to cell transformation or cancer progression phenotype seem to be a general feature of mammary cell exposed to estrogens or xenoestrogens.

## Ethics Statement

All experimental procedures were approved by the French Minister of Research Committee for animal experiment in accordance with the Guide for Care and Use of Laboratory Animals. The transgenerational model of C57BL/6J exposed to alkylphenols was carried out in accordance with the recommendations of French Minister of Research Committee for animal experiment (Protocol no. APAFIS#2168-2015110518268051 v5). Cell grafting in Nude mice exposed to alkylphenols was carried out in accordance with the recommendations of French Minister of Research Committee for animal experiment (Protocol no. APAFIS#6158-2016072016446146 v5).

## Author Contributions

Conceptualization: HD, AC, and TB. Data curation: EB and TB. Formal analysis: AC, HD, M-DD, and TB. Funding acquisition: HD and TB. Investigation: CT, CC-J, and CM. Methodology: CT, CC-J, AC, and MS-T. Project administration: AC, TB, and HD. Resources: AC, CC-J, and HD. Software: EB, MS-T, M-DD, and TB. Supervision: HD, TB, and AC. Validation: AC, HD, and TB.

## Conflict of Interest Statement

The authors declare that the research was conducted in the absence of any commercial or financial relationships that could be construed as a potential conflict of interest.

## Footnotes

^1^http://pantherdb.org.

^2^http://www.broadinstitute.org/gsea/msigdb/.
