# Supplementary material for: Low-Dose Alkylphenol Exposure Promotes Mammary Epithelium Alterations and Transgenerational Developmental Defects, But Does Not Enhance Tumorigenic Behavior of Breast Cancer Cells
Source: Front Endocrinol (Lausanne). 2017 Oct 23;8:272. doi: 10.3389/fendo.2017.00272 (PMC5660105; doi:10.3389/fendo.2017.00272)
Supplement: Supplementary file 1 [file Table_1.PDF]

Supplementary Table 1

A

GO biological process	Fold Enrichment t = 1 h		GO biological process	Fold Enrichment t = 1h
response to stress	1,83		regulation of primary metabolic process	1,51
cellular nitrogen compound metabolic process	1,63		regulation of cellular metabolic process	1,5
organic cyclic compound metabolic process	1,57		primary metabolic process	1,37

B

GO biological process	Fold Enrichment t = 24 h		GO biological process	Fold Enrichment t = 24 h
DNA strand elongation involved in DNA replication	8,09		response to ionizing radiation	2,54
kinetochore organization	7,85		nucleocytoplasmic transport	2,51
CENP-A containing nucleosome assembly	7,30		positive regulation of DNA metabolic process	2,44
nuclear DNA replication	6,92		nucleic acid phosphodiester bond hydrolysis	2,43
telomere maintenance via recombination	6,73		mRNA splicing, via spliceosome	2,28
DNA replication initiation	6,68		negative regulation of cell cycle phase transition	2,23
spindle checkpoint	5,88		negative regulation of organelle organization	2,08
mitotic metaphase plate congression	5,74		cell proliferation	1,97
DNA damage response, detection of DNA damage	5,56		regulation of cellular response to stress	1,91
regulation of transcription involved in G1/S transition of mitotic cell cycle	5,46		regulation of cellular amide metabolic process	1,82
sister chromatid cohesion	5,37		posttranscriptional regulation of gene expression	1,8
DNA-dependent DNA replication maintenance of fidelity	4,94		regulation of cellular protein localization	1,76
chromosome condensation	4,94		protein complex assembly	1,62
regulation of mitotic metaphase/anaphase transition	4,81		cellular macromolecule catabolic process	1,57
regulation of DNA-dependent DNA replication	4,76		organonitrogen compound catabolic process	1,53
mitotic spindle assembly	4,71		cell death	1,51
microtubule polymerization or depolymerization	4,41		small molecule metabolic process	1,44
regulation of cholesterol biosynthetic process	4,20		regulation of cell proliferation	1,43
DNA synthesis involved in DNA repair	3,77		response to oxygen-containing compound	1,42
positive regulation of cell cycle phase transition	3,60		negative regulation of cellular biosynthetic process	1,41
regulation of DNA recombination	3,53		regulation of multicellular organismal development	1,39
cell division	3,52		negative regulation of macromolecule metabolic process	1,39
DNA geometric change	3,32		negative regulation of nitrogen compound metabolic process	1,38
double-strand break repair via homologous recombination	3,32		regulation of cellular protein metabolic process	1,36
G2/M transition of mitotic cell cycle	2,98		multi-organism process	1,35
positive regulation of mitotic cell cycle	2,92		regulation of catalytic activity	1,31
mitotic DNA damage checkpoint	2,91		animal organ development	1,3
meiotic nuclear division	2,76		G-protein coupled receptor signaling pathway	0,39
regulation of cell division	2,74		sensory perception of smell	<0,2
regulation of microtubule cytoskeleton organization	2,74		detection of chemical stimulus involved in sensory perception	<0,2
regulation of signal transduction by p53 class mediator	2,63			

Supplementary Table 1. Gene Ontology enrichment analysis of differentially expressed genes after a 1 h or 24 h 1nM M4 exposure.

Biological process GO terms significantly (p-value <0.05) enriched after 1h (A) or 24h (B) alkylphenol exposure and respective Fold enrichment were obtained using the PANTHER database (Protein ANalysis THrough Evolutionary Relationships, http://pantherdb.org).

The overlap between the 76 DEGs obtained after 8 h M4 exposure and GO gene sets derived from the Biological Process didn’t highlight GO terms significantly enriched at this point of the kinetic.
