# Supplementary material for: Low-Dose Alkylphenol Exposure Promotes Mammary Epithelium Alterations and Transgenerational Developmental Defects, But Does Not Enhance Tumorigenic Behavior of Breast Cancer Cells
Source: Front Endocrinol (Lausanne). 2017 Oct 23;8:272. doi: 10.3389/fendo.2017.00272 (PMC5660105; doi:10.3389/fendo.2017.00272)
Supplement: Supplementary file 2 [file Table_2.PDF]

Supplementary Table 2

1 hour		8 hours		24 hours	
KEGG	Genes in overlap	KEGG	Genes in overlap	KEGG	Genes in overlap
KEGG_PATHWAYS_IN_CANCER	7	KEGG_PATHWAYS_IN_CANCER	4	KEGG_PATHWAYS_IN_CANCER	41
KEGG_FOCAL_ADHESION	6	KEGG_CELL_CYCLE	4	KEGG_CELL_CYCLE	37
KEGG_REGULATION_OF_ACTIN_CYTOSKELETON	6	KEGG_DNA_REPLICATION	3	KEGG_SPLICEOSOME	31
KEGG_MAPK_SIGNALING_PATHWAY	6	KEGG_P53_SIGNALING_PATHWAY	3	KEGG_PYRIMIDINE_METABOLISM	26
KEGG_JAK_STAT_SIGNALING_PATHWAY	5	KEGG_JAK_STAT_SIGNALING_PATHWAY	3	KEGG_DNA_REPLICATION	25

Supplementary Table 2. Pathway enrichment analysis of differentially expressed genes after a 1 h, 8 h or 24 h 1nM M4 exposure.

KEGG pathways significantly (p-value <0.05) enriched after 1h, 8 h or 24h alkylphenol were obtained using the MSigDBv5.0 database (Molecular Signature Database http://www.broadinstitute.org/gsea/msigdb/).

At each point of the kinetics, the maximal number of genes in overlap is obtained for the KEGG Pathway “Pathways in cancer”.
