# Supplementary material for: Low-Dose Alkylphenol Exposure Promotes Mammary Epithelium Alterations and Transgenerational Developmental Defects, But Does Not Enhance Tumorigenic Behavior of Breast Cancer Cells
Source: Front Endocrinol (Lausanne). 2017 Oct 23;8:272. doi: 10.3389/fendo.2017.00272 (PMC5660105; doi:10.3389/fendo.2017.00272)
Supplement: Supplementary file 3 [file Presentation_1.PDF]

Supplementary Figure 1

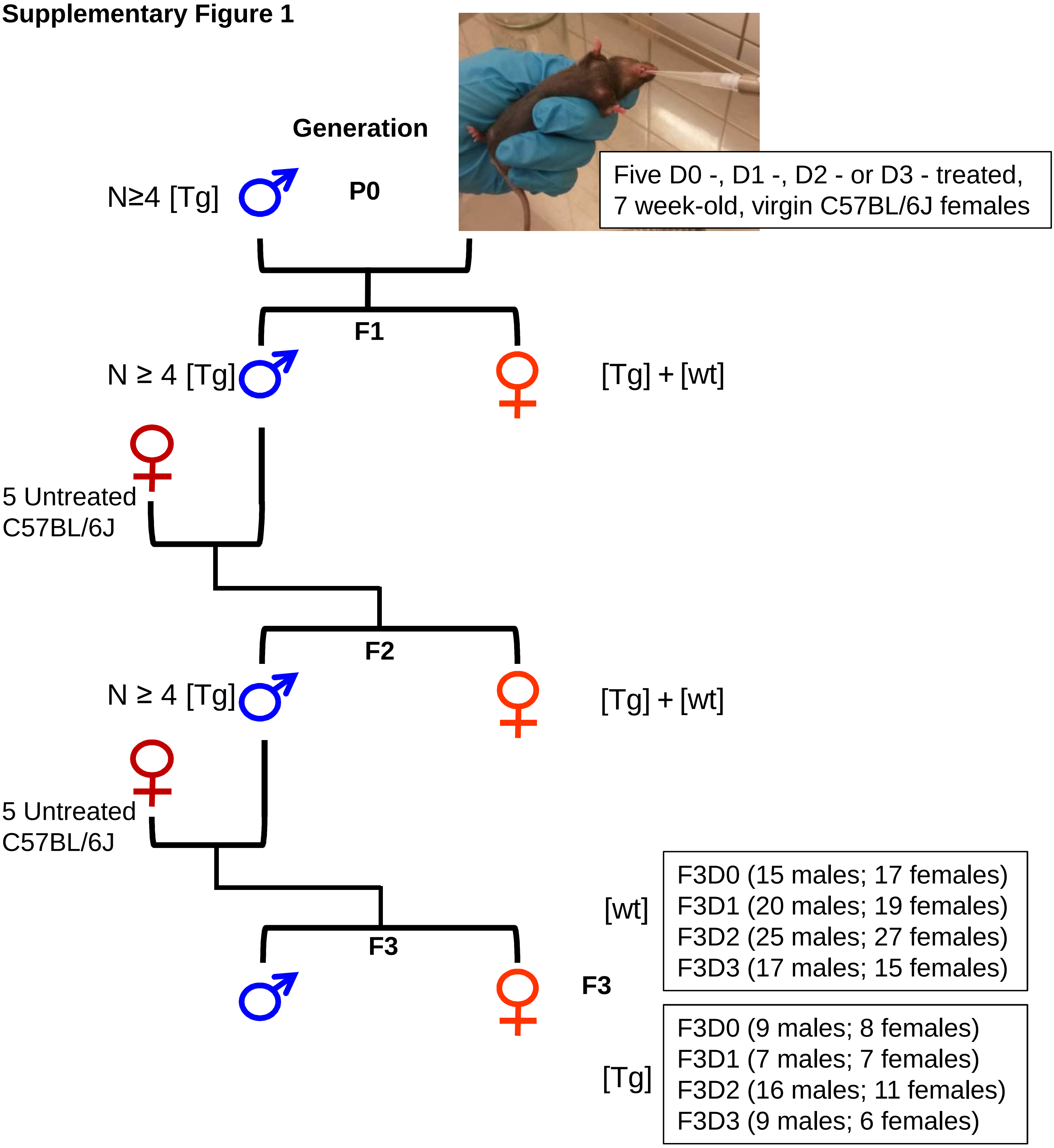

Supplementary Figure 1: Male inherited transgenerational model of exposure in ERα36 transgenic mouse. Previously described (16) ERα36 hemizygotic transgenic (Tg) males were mated with P0 C57BL/6J (wt) virgin adult females treated with M4 dissolved in vehicle (0.05µg/kg/day (D1), 0.5µg/kg/day (D2) or 5µg/kg/day (D3) or either sesame oil (vehicle, D0). See materials and methods section for details. Hemizygotic F1 transgenic males were selected as previously described (16) and at least 4 of them, issued from D0 to D3 maternal treatment, were mated with 5 or more wt adult virgin females. The same experiment was repeated with F2 transgenic males. The male inherited transgenerational effect of the 3 different doses of alkyphenol treatment was observed in the mammary gland from F3 progenies. Neither the transgene nor the treatments affected significantly the sex ratio whereas we observed a statistically significant deficiency of transgenic animals (-9% and -8% transgenic males and females respectively; p= 0.009) under alkylphenol exposure whatever the dose of M4 given to P0 dams.

Supplementary Figure 2

A

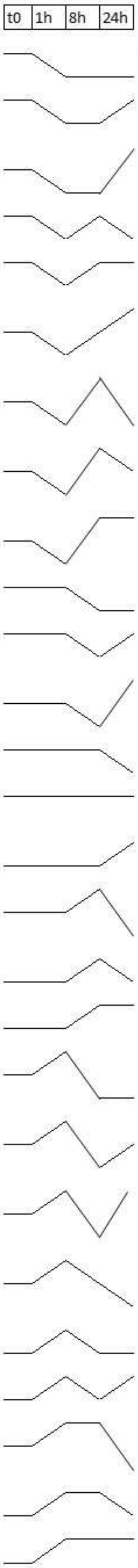

Variation pattern	Gene number
-1-1-1	232
-1-10	41
-1-11	2
-10-1	92
-100	124
-101	4
-11-1	0
-110	0
-111	0
0-1-1	349
0-10	173
0-11	3
00-1	588
000	38,329
001	1,188
01-1	1
010	100
011	200
1-1-1	0
1-10	0
1-11	0
10-1	1
100	56
101	71
11-1	0
110	15
111	122
TOTAL	41,691

B

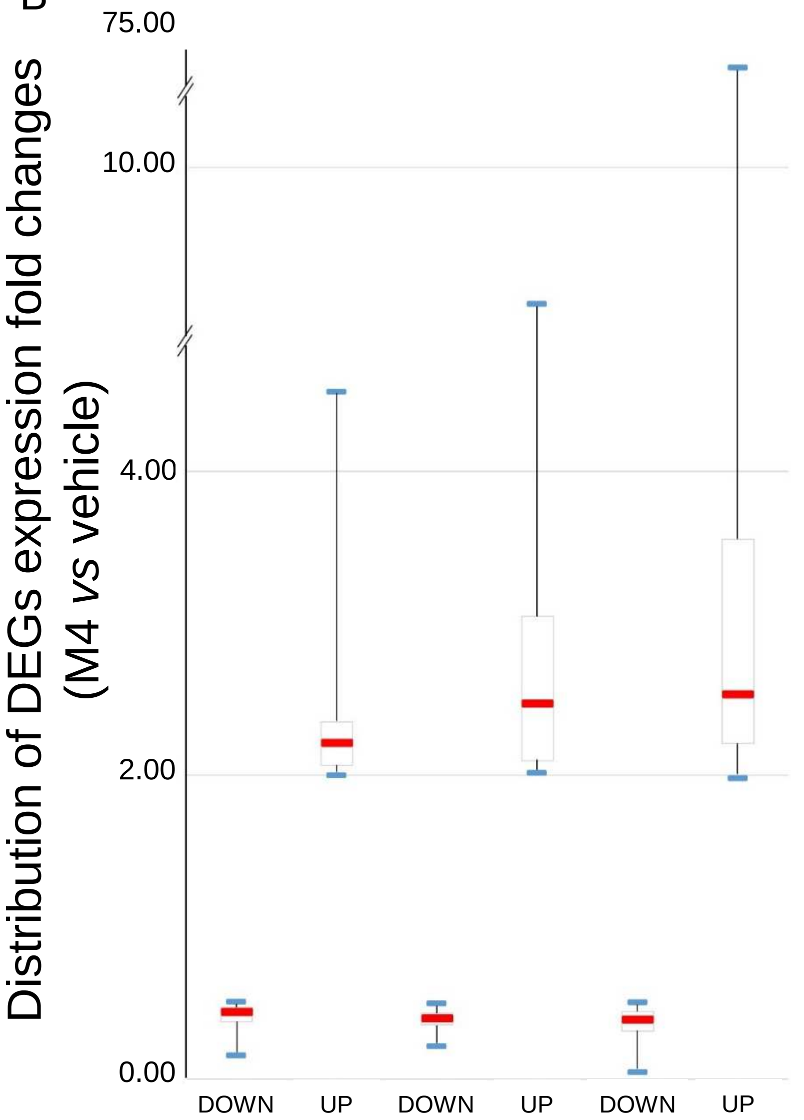

DEG number	156	122	34	42	1,211	1,532
	278		76		2,743	
	**M4 1h**		**M4 8h**		**M4 24h**	

Supplementary Figure 2. DEG transcriptional profiles and distribution after a kinetic of MCF-10A cell exposure to alkylphenol mixture. A) Kinetic variation pattern of MCF-10A genes after alkylphenol exposure. The variation of expression is evaluated at 1 hour, 8 hours and 24 hours of treatment versus control. If genes expression variation is under 0.5, index of variation is set at -1 (down-regulated genes). If genes expression variation is over 2, index variation is set at 1 (up-regulated genes). Between 0.5 and 2, gene expression variation is considered non-significantly changed, index variation is set at 0. Gene number in each variation pattern is indicated in the right column. **B)** Boxplots indicating the distribution of up- or down-regulated DEG expression fold changes (M4 1nM *vs* vehicle) for each kinetic time point.

Supplemental Figure 3

A

Gene name	Fold expression variation 24h-M4/Veh
MCM2	6.85
MCM3	8.77
MCM4	5.91
MCM5	5.19
MCM6	2.11
MCM8	2.51
MCM10	13.98
BRCA1	2.41
MSH2	2.6
MSH6	2.3
RAD51	9.67

B

RAPD primer	Sequence	GC content (%)
RAPD 1	CAGGCCCTTC	70
RAPD 2	TGCCGAGCTG	70
RAPD 3	AGTCAGCCAC	60
RAPD 4	AATCGGGCTG	60
RAPD 5	AGGGGTCTTG	60
RAPD 6	GGTCCCTGAC	70
RAPD 7	GAAACGGGTG	60
RAPD 8	GTGACGTAGG	60
RAPD 9	GGGTAACGCC	70
RAPD 10	GTGATCGCAG	60

C

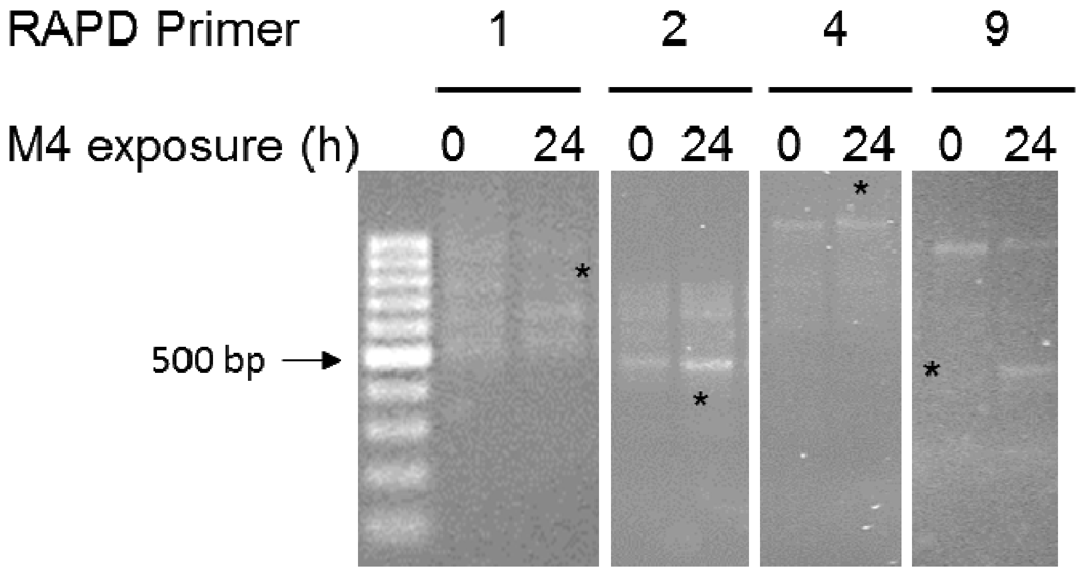

Supplementary Figure 3. M4 alters genomic stability of MCF-10A cells. A) Fold expression variation after 24h 1nM M4 exposure of DEGs assigned to “Failed repair of genes”. **B)** RAPD primer sequences and GC base contents. **C)** RAPD profiles (26, 27). Ten random GC-rich decanucleotide primers were used to score genomic alterations of MCF-10A cells exposed 24h to 1nM M4 or vehicle. Each genomic DNA pool was amplified with each individual primer. The random amplified polymorphic DNA (RAPD) reaction was performed in a volume of 15µL containing 7.5µL iQ SYBR Green Supermix (Biorad), 0.3µM primer and 20 ng genomicDNA. Cycling parameters were as follows: 95°C for 5 minutes, 40 cycles (95°C for 30 seconds, 37°C for 30 seconds, and 72°C for 1 minute). Polymerase chain reaction (PCR) products were resolved by electrophoresis on a 2% tris-acetate-EDTA–agarose gel and visualized by ethidium bromide staining (Geldoc, Biorad). Each experiment was repeated three times. Samples that display loss, addition or significant fluorescence intensity variation of the banding profile are marked (*) and termed as « genomic unstable ». The frequency of altered RAPD profile is about 50%.

Supplementary Figure 4

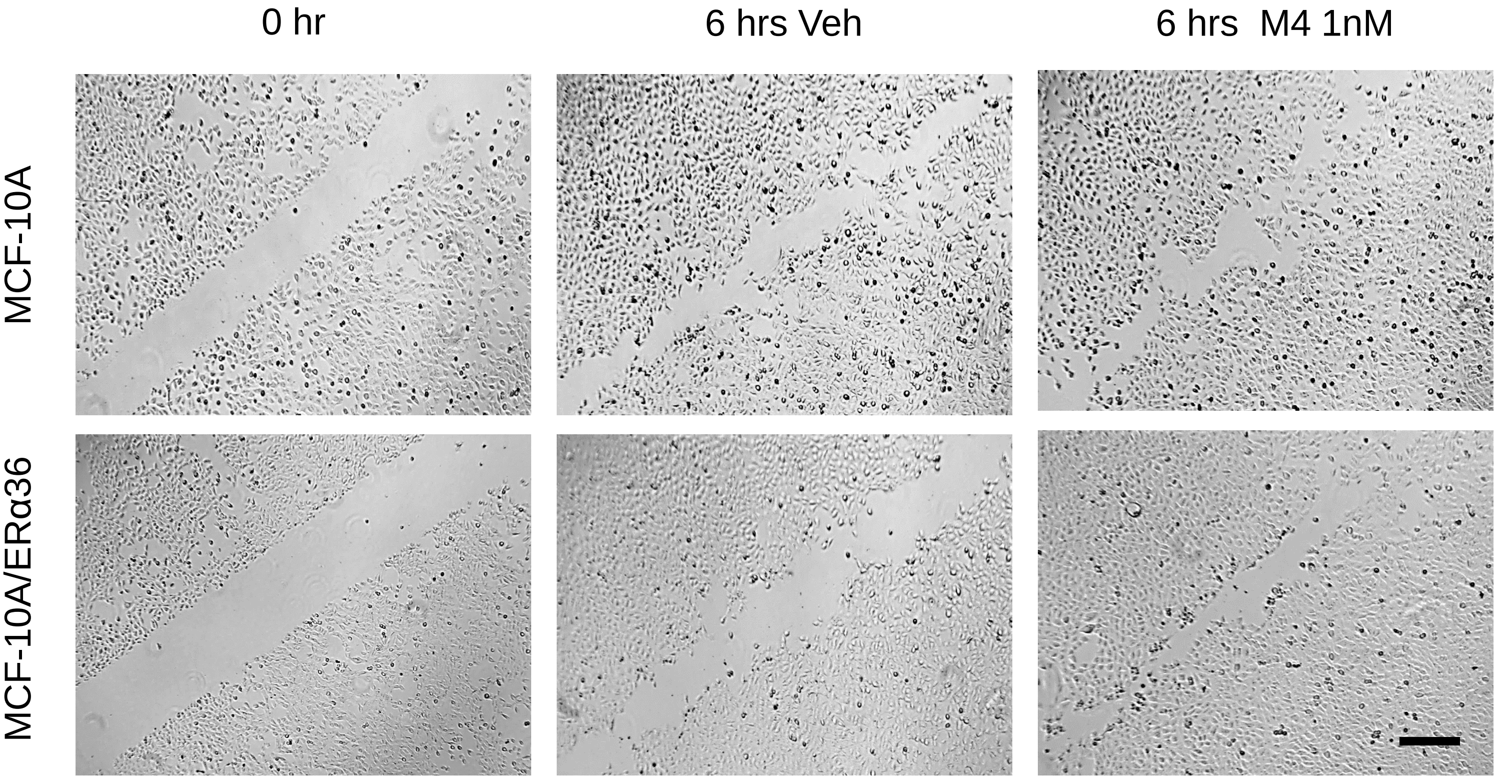

Supplementary Figure 4. Representative images of scratch wound assays. Scratch assays were performed on MCF-10A and MCF-10A/ERα36 cultures using the Ibidi-culture inserts (Ibidi®/Biovalley) following the manufacturer instructions. Cultures were then washed to remove detached cells and debris and treated with vehicle or M4. Quantification of wound mean diameter were performed at t=0 and after a 6 h exposure by Phase-contrast image analysis with NIS-elements BR 4.20.00 software (Nikon). Scale bar: 1mm.

Supplementary Figure 5

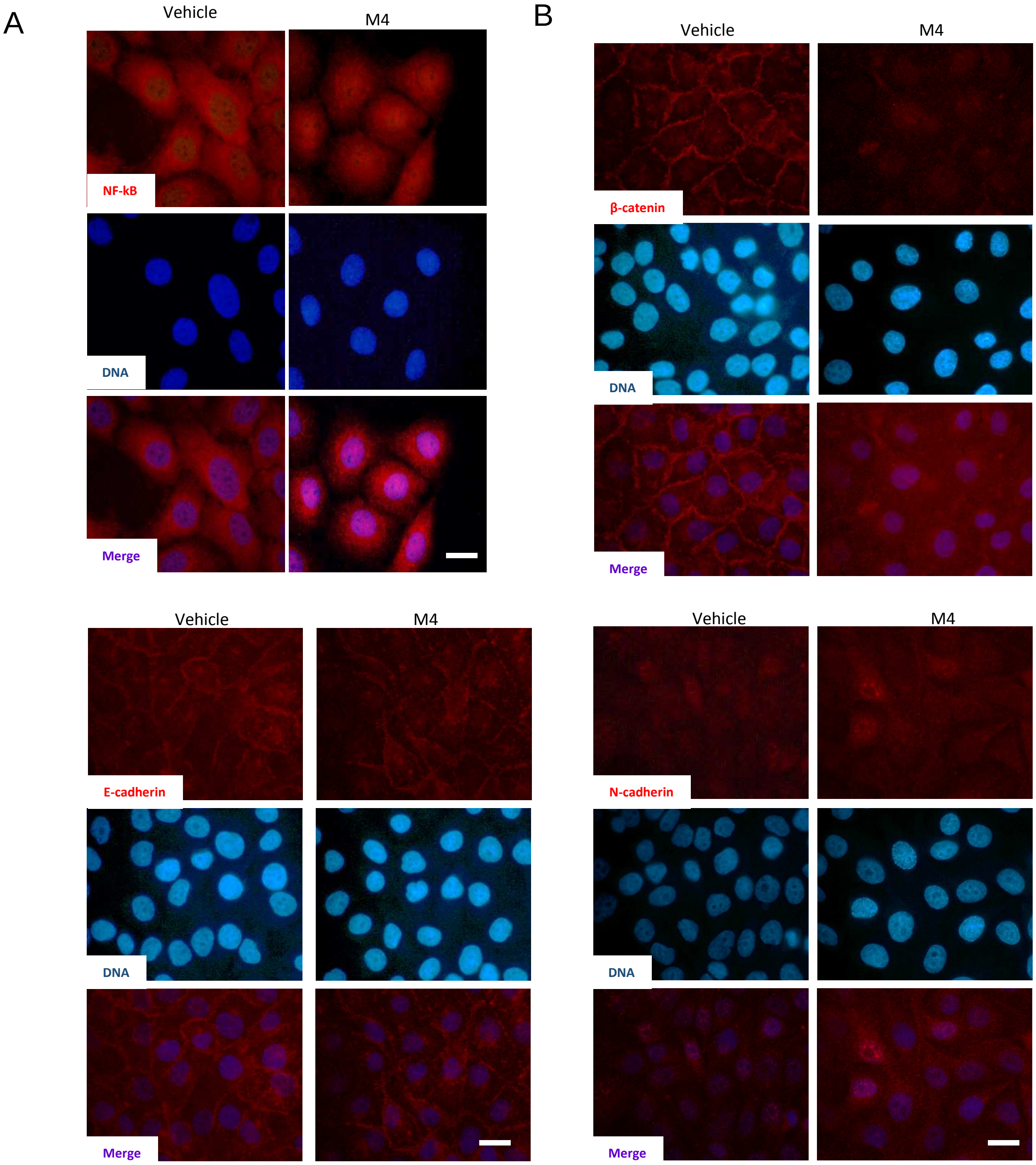

Supplementary Figure 5. Cellular relocation of NF-κB, β-catenin, E-cadherin, N-cadherin and ZO-1after M4 treatment. MCF-10A cells were treated for 24 h with vehicle or 1nM M4. Localization of NF-κB and cell-cell adhesion proteins were performed by immunofluorescence with specific antibodies (red, AlexaFluor 555). Hoechst was used to stain the cell nuclei (blue). **A)** NF-κB nuclear translocation is observed in MCF-10A cells exposed to M4. **B)** A decrease of beta-catenin membrane immunofluorescence staining toward a dispersed cytoplasmic localization and an "E-to N-cadherin switch" are observed in M4-treated cells compared to control. Scale bar: 5μm.

Supplementary Figure 6

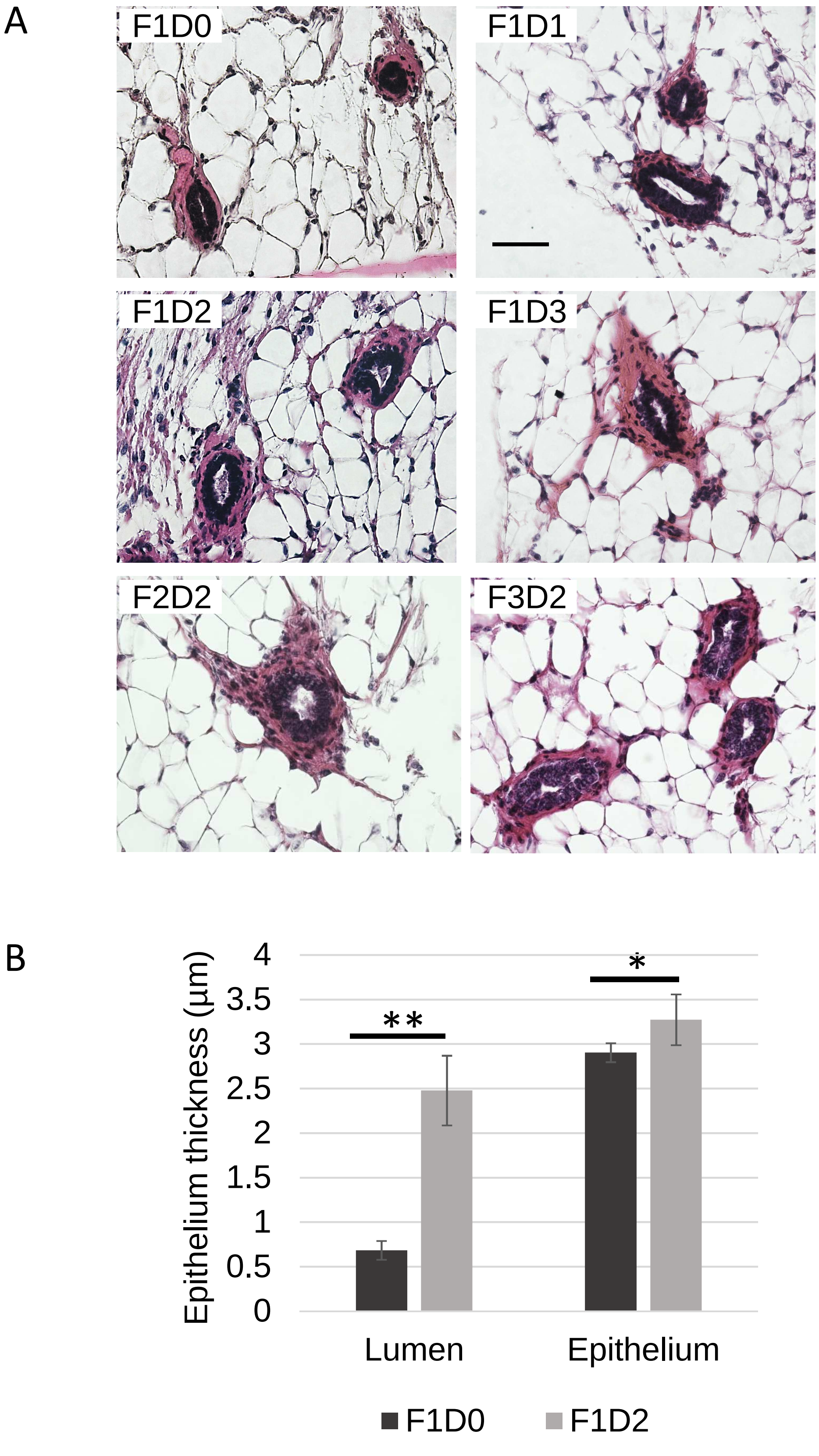

Supplementary Figure 6. Hematoxylin/Eosin staining of mammary gland at weaning (PND 21). A) Representative histology of F1 mammary gland exposed to vehicle (D0) or M4 (D1= 0.05µg/kg/day, D2=0.5µg/kg/day, D3=5µg/kg/day)as well as F2D2 and F3D2 mammary glands. **B)** Corresponding quantification of lumen diameter and epithelium thickness for F1D0 and F1D2 mammary glands. A significant increase in all measured parameters is observed in D2 exposed animals (N=5) compared to D0 ones (N=8). Scale bar, 10 µm. Each bar represents mean ± S.E.M. All parameters were measured on at least 5 independent slices from each animal included in the experiment. *: $p<0.05$; **: $p< 0.01$.

Supplementary Figure 7

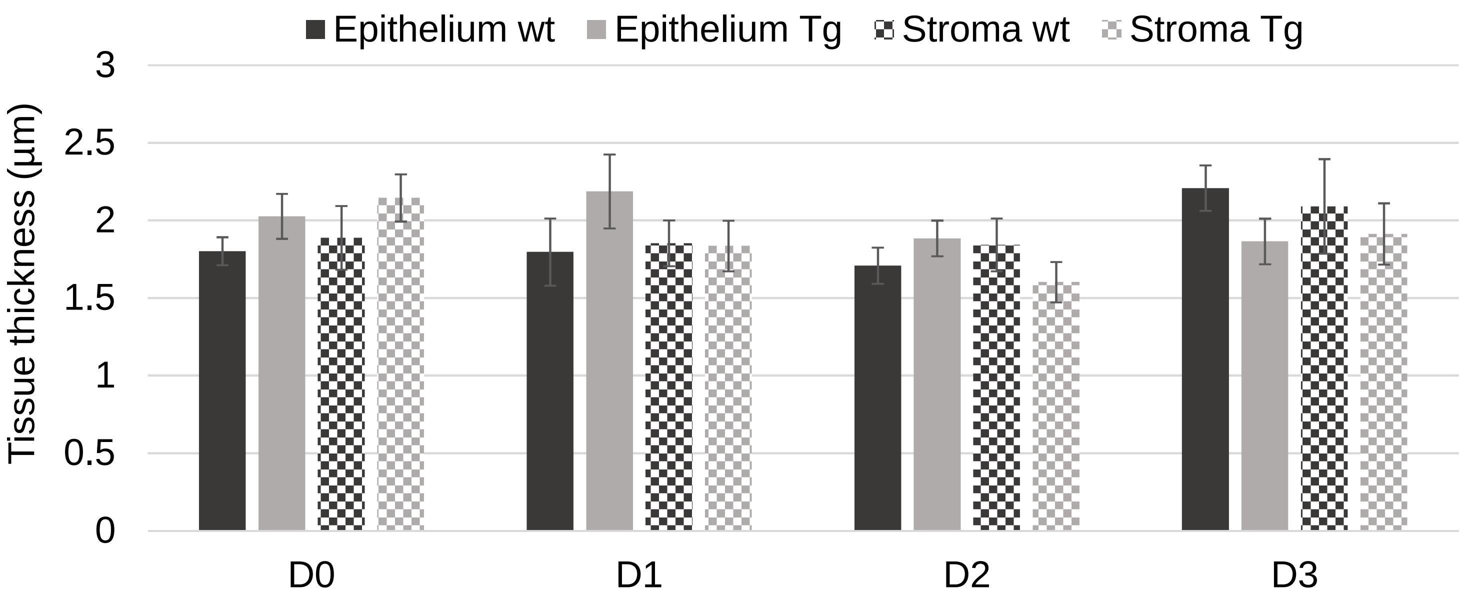

Supplementary Figure 7. Quantification of epithelium and stroma thickness in F3 mammary gland following exposure to sesame oil (D0) or M4 (0.05µg (D1), 0.5µg (D2) or 5µg M4/kg/day (D3)). Measurements were performed at adulthood (16 weeks). Each bar represents mean ± S.E.M. Epithelium or stroma thickness was measured on at least 5 independent slices from each animal included in the experiment. N≥ 3.

Supplementary Figure 8

(A)

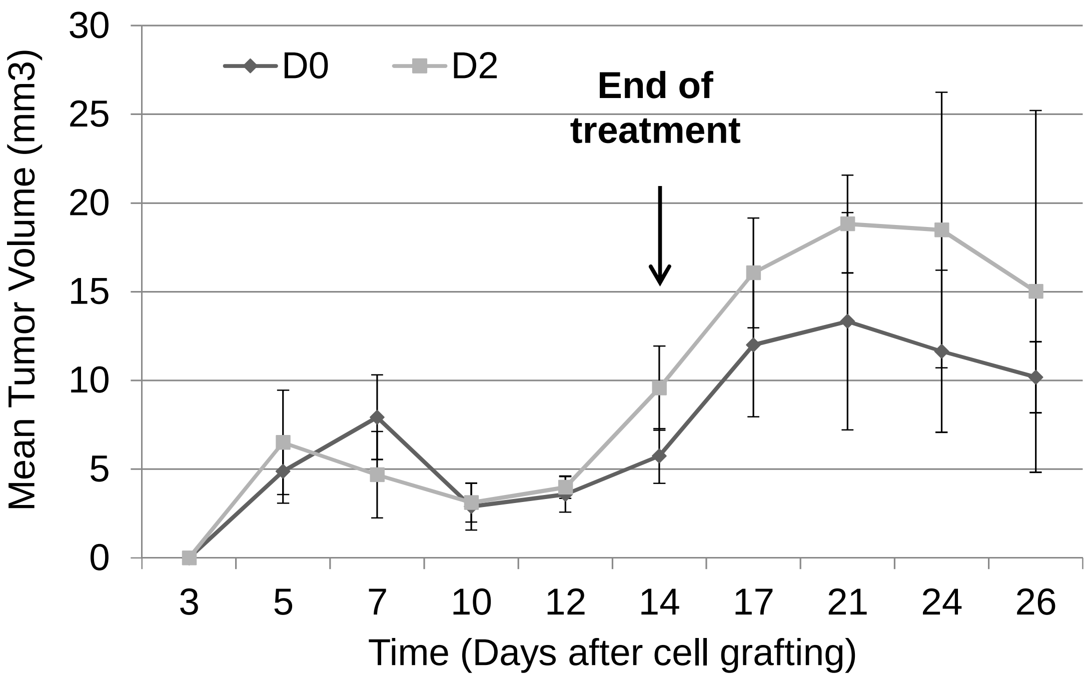

(B)

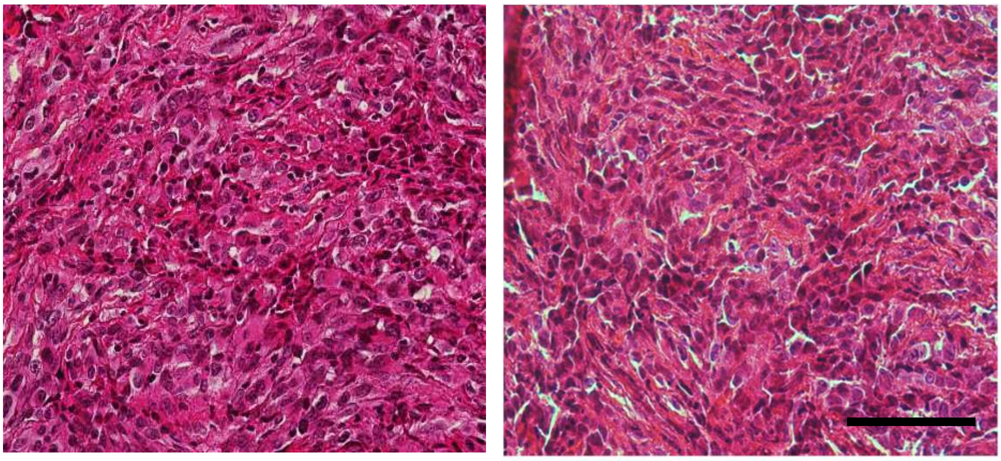

Supplementary Figure 8 : Effects of M4 exposure on tumor growth in nude mice xenografted with MDA-MB-231 breast cancer cells.

As described in the Materials and Methods section, D0 and D2 treatments were administered by intrabuccal gavage, during two weeks before and two weeks after cell grafting in the right mammary gland no.4 of nude mice.

(A) Tumor growth was measured during four weeks after MDA-MB-231 cells injection. In each group (D0 and D2), one mouse did not develop a tumor. For each point of the kinetics the mean tumor volume (mm^3) ± S.E.M was indicated. N=4 per dose.

(B) Hematoxylin/Eosin staining of D0 and D2 mammary tumors collected four weeks after cell grafting. Scale bar, 50 µm.

No significant differences were observed concerning tumor growth kinetics and tumor histology between the 2 groups.
